# Conjugated Bisphosphonic Acid Self-Assembled Monolayers for Efficient and Stable Inverted Perovskite Solar Cells

**DOI:** 10.1021/jacs.5c05801

**Published:** 2025-06-30

**Authors:** Songyang Yuan, Chengda Ge, Tianyi Zhang, Gengyang Su, Quanrun Qiu, Guanhua Ren, Lingyi Ke, Gengxin Du, Guangruixing Zou, Nan Zhang, Hui Liu, Qingduan Li, Tao Jia, Yue-Peng Cai, Shengjian Liu, Hin-Lap Yip

**Affiliations:** † School of Chemistry, Guangzhou Key Laboratory of Materials for Energy Conversion and Storage, Key Laboratory of Electronic Chemicals for Integrated Circuit Packaging, South China Normal University (SCNU), Guangzhou 510006, China; ‡ Department of Materials Science and Engineering, 53025 City University of Hong Kong, Kowloon, Hong Kong 999077, China; § Guangdong Zenithnano New material Co., Ltd., Guangzhou 510006, China; ∥ School of Mechanical Engineering, Guangdong Polytechnic Normal University, Guangzhou 510665, China; ⊥ Hong Kong Institute for Clean Energy (HKICE), 53025 City University of Hong Kong, Kowloon, Hong Kong 999077, China; # State Key Laboratory of Marine Pollution, 53025 City University of Hong Kong, Kowloon, Hong Kong 999077, China; ∇ School of Energy and Environmental Science, 53025 City University of Hong Kong, Kowloon, Hong Kong 999077, China

## Abstract

Chemically modifiable self-assembled monolayer (SAM)-based hole transport layers are crucial for achieving high-efficiency inverted perovskite solar cells (PSCs). However, designing molecular structures that simultaneously ensure strong binding affinity, interfacial stability, and optimized energy level alignment remains challenging. Here, we introduce TPA2P ((2-(4-(diphenylamino)­phenyl)-1-phosphonovinyl)­phosphonic acid), a novel SAM material featuring a conjugated bisphosphonic acid anchoring group. This dual phosphonic acid configuration enhances substrate binding on indium tin oxide (ITO), improves SAM uniformity, and increases interfacial stability. Furthermore, the ethylene bridge facilitates efficient intramolecular charge transfer (ICT) from the electron-donating triphenylamine unit to the electron-accepting bisphosphonic acid group. This ICT induces significant charge redistribution in TPA2P, resulting in a deep HOMO level at −5.47 eV. This optimized energy alignment reduces interfacial energy losses and significantly enhances hole extraction efficiency. As a result, TPA2P-based inverted PSCs achieve a high power conversion efficiency of 26.11%, an exceptional fill factor of 85.03%, and outstanding operational stability under continuous illumination. These findings provide an effective molecular design strategy for advancing high-performance and stable perovskite photovoltaics.

## Graphical abstract

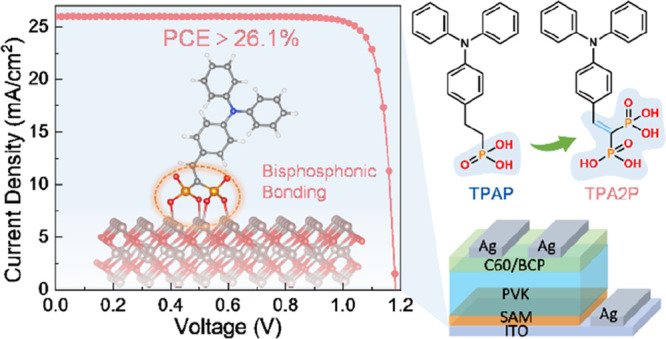

## Introduction

Inverted perovskite solar cells (PSCs) that use self-assembled monolayers (SAM) as hole transport layers (HTLs) have made significant progress in recent years, achieving record power conversion efficiencies (PCE) exceeding 26%.^1–3^ However, the practical implementation of SAM still faces persistent challenges, particularly due to the weak binding affinity of SAM molecules to metal oxide substrates (e.g., indium tin oxide, ITO) and their propensity for nonuniform deposition.^4–7^ These molecular-level imperfections, such as incomplete surface coverage and random molecular orientation, exacerbate interfacial nonradiative recombination and increase charge transport losses. As a result, these issues limit further improvements in both the efficiency and long-term stability of PSCs.^8^

The interaction between SAM molecules and metal oxide substrates is primarily controlled by anchoring groups, which determine how well SAMs attach to the substrate. The strength of this interaction plays a crucial role in ensuring uniform SAM coverage and maintaining high operational stability, both of which are key factors affecting overall device performance.^7–10^ To address these challenges, researchers have explored various molecular engineering strategies.

One effective approach involves modifying the solvent environment to improve SAM deposition. For example, mixed-solvent systems have been utilized to prevent SAM molecules from aggregating and to promote more uniform film formation. In a pioneering study, Jen et al. demonstrated that a methanol/chlorobenzene cosolvent system could effectively break down micellar aggregates of carbazole-based SAMs, leading to nearly complete substrate coverage.^11^ Another promising strategy is molecular cross-linking, which enhances the adhesion of SAMs to the substrate.^8,12^ Chen and co-workers developed a polymer-based hole-transporting SAM material, Poly-DCPA, which exhibited excellent conductivity and uniformity. This polymeric SAM not only improved interfacial adhesion with ITO but also contributed to greater device stability.^8^ Additionally, researchers have explored mixed SAMs and using SAM as additives as a way to reduce molecular aggregation and improve film coverage.^7,13,14^ For instance, Li et al. introduced methylene blue as a coadsorbent alongside Me-4PACz. By leveraging π–π interactions, they were able to create a more uniform SAM distribution, eliminate pinholes, and form a compact interfacial layer, ultimately enhancing the stability of the photovoltaic device.^15^

Despite advances have been made in SAM-based HTLs through terminal group modifications, mixed SAM systems, and solvent polarity adjustments, comprehensive studies on anchoring groups, such as extending conjugated systems and using multidentate strategies, are still evolving.^16–18^ These approaches have shown promise in enhancing interfacial interactions, but systematic design principles and their precise impact on device performance warrant further detailed exploration. Although monophosphonic acid anchoring groups are widely used due to their decent binding properties on ITO, they may not fully exploit the potential for stronger interfacial adhesion and long-term device stability.

To address this limitation, we introduce a conjugated bisphosphonic acid-functionalized SAM design, employing (2-(4-(diphenylamino)­phenyl)-1-phosphonovinyl)­phosphonic acid (TPA2P) as the HTL. The dual phosphonic acid anchoring groups in TPA2P facilitate multidentate chelation with ITO, resulting in significantly stronger binding affinity compared to conventional monophosphonic analogs, thereby improving both SAM layer coverage and stability. Concurrently, strong electron-withdrawing group not only modulates the molecular dipole but also effectively deepens the HOMO energy level.^19^ The introduction of the additional phosphate through the conjugated ethylene linker, compared to a single phosphate group, enhances the electron-withdrawing effect. Therefore, comparing the more commonly used carbazole group, the SAM synthesized with triphenylamine as the terminal group may similarly achieve favorable HOMO level alignment with the perovskite layer. This refined HOMO level facilitates better energy level alignment for hole extraction and minimizes photovoltage losses and contributes to improved device performance.

Additionally, the rigid conjugated backbone suppresses molecular torsion, facilitating better crystallization of the overlying perovskite film, which is an essential factor for optimizing charge transport. As a result, this molecular design strategy achieves an exceptional fill factor (FF) of 85.03%, an open-circuit voltage (*V*_OC_) exceeding 1.18 V, and a short-circuit current density (*J*_SC_) of 25.99 mA cm^–2^, leading to an enhanced PCE of 26.11%—an improvement of nearly 10% compared to the TPAP-based device. Moreover, the reinforced interfacial adhesion significantly enhances operational stability, with unencapsulated devices retaining 96.7% of their initial PCE after 1000 h of continuous illumination under a nitrogen atmosphere. The following sections provide a comprehensive investigation into these structure–function relationship, device performance, and long-term stability, offering critical insights into the rational design of next-generation inverted perovskite solar cells.

## Results

### Molecular Design of TPA2P

The synthetic route of TPA2P is depicted in Scheme S1, and the corresponding structural characterization data are provided in the Supporting Information section (Figures S1 and S2). The chemical structures and purity of these compounds were confirmed using ^1^H Nuclear Magnetic Resonance (NMR) spectroscopy.

Figure 1a illustrates the molecular structures of TPA2P and (2-(4-(diphenylamino)­phenyl)­ethyl)­phosphonic acid (TPAP), both featuring a triphenylamine (TPA) terminal group as the electron-donating unit and TPAP is designed as a control material. Compared to TPAP, the TPA2P incorporates two strategic modifications of its molecular structure: (i) a bisphosphonic acid acting as a stronger electron-accepting motif and anchoring group, and (ii) a conjugated ethylene linker bridging the TPA core and the anchoring moieties. The double bond connection facilitates a pronounced conjugation and inductive effect. Meanwhile, the second phosphonic acid group is anticipated to increase the intrinsic dipole moment and enhance binding affinity with ITO. Collectively, these structural modifications are expected to promote surface coverage, lower the HOMO energy levels, and strengthen interfacial dipole interactions, thereby improving overall device performance.

**Figure 1:**
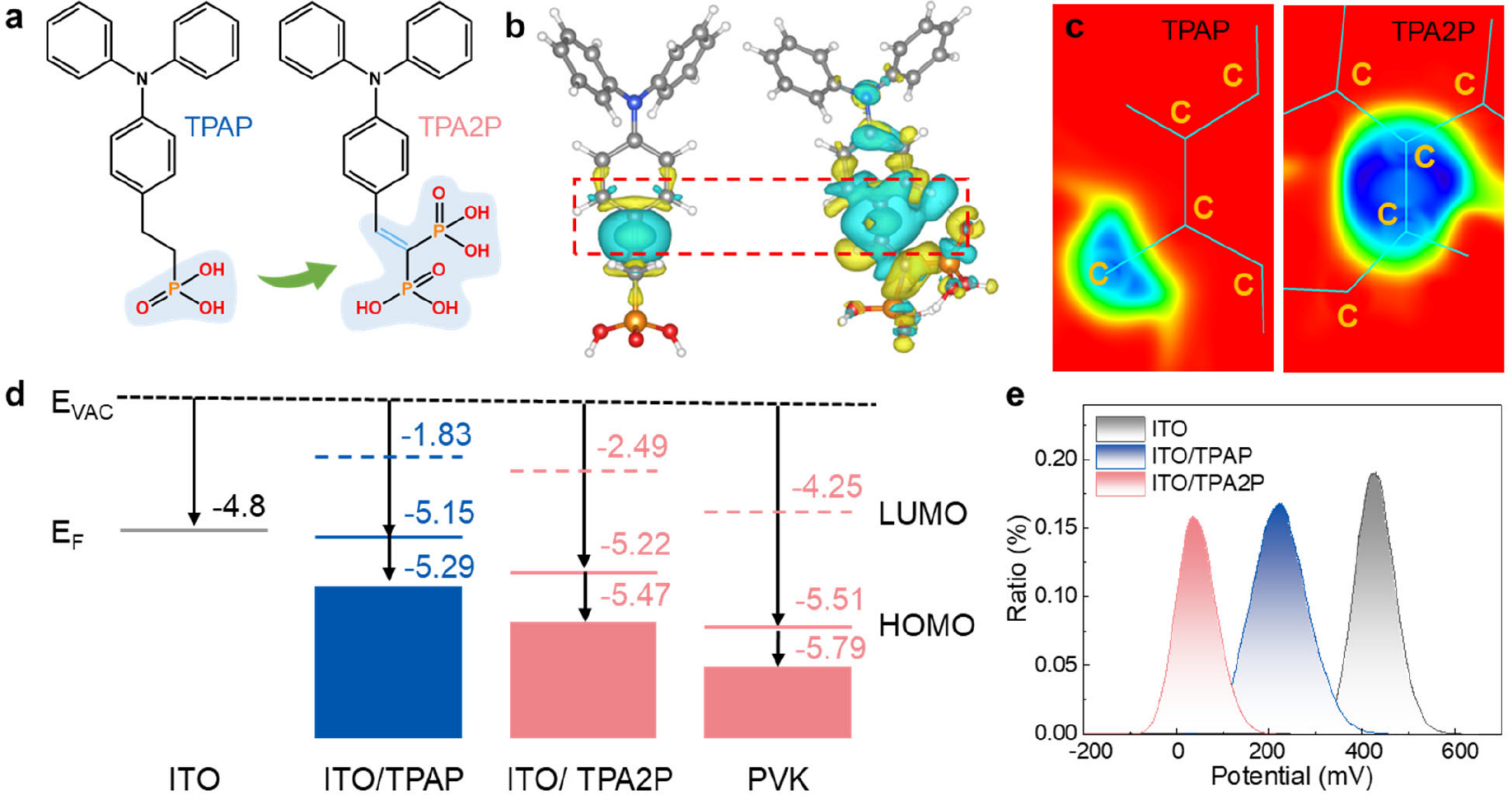
(a) Chemical structures of TPAP and TPA2P. (b) Intramolecular charge density differences of TPAP and TPA2P. the electron depletion/accumulation is depicted by the blue/yellow isosurfaces. at ± 0.002 |e| Å^–3^. (c) Local ELF comparison diagram of TPAP and TPA2P. (Red dashed-line boxed area in Figure 1b) (d) Schematic representation of the band edge distributions of the SAM and perovskite layers, based on UPS values referenced to the vacuum level. The *E*_F_ and *E*_VAC_ represent Fermi and vacuum levels, respectively. (e) Statistical distribution of surface potentials for bare ITO substrate and ITO substrates covered by TPAP and TPA2P.

As evidenced by the intramolecular charge density differences and electron localization function (ELF) maps (Supplementary Note 1, Figure 1b,c), the bisphosphonic acid groups in TPA2P exhibit enhanced electron-withdrawing character. This synergizes with the conjugated double bond to amplify electron delocalization across the molecule. This electronic modulation is further quantified by the electrostatic surface potential (ESP) and molecular dipole moments (Figure S3). Notably, TPA2P displays a significantly larger dipole moment (4.87 D) compared to TPAP (1.69 D). Moreover, the bisphosphine structure was expected to give a stronger interaction with ITO, it facilitates a more ordered molecular structure and dipole moment arrangement, offering an enhanced ability to modulate interfacial energy levels and improve charge extraction properties, contingent upon the precise orientation of the dipole at the interface.^20,21^

As shown in Figure S4, TPA2P demonstrates a red-shifted absorption edge relative to TPAP, with optical bandgaps estimated at 3.46 eV for TPAP and 2.98 eV for TPA2P. This bandgap reduction is attributed to the inductive effect of electron-withdrawing bisphosphonic acid groups and the conjugated ethylene linker, which collectively lower the energy levels of TPA2P through enhanced charge delocalization. Density functional theory (DFT) calculations reveal that TPA2P exhibits a deeper HOMO level at −5.66 eV compared to TPAP (−5.38 eV), consistent with their differences in intramolecular charge density profile (Figure 1b). Notably, the lowest unoccupied molecular orbital (LUMO) analysis reveals a stronger electron density localization around the bisphosphonic acid groups in TPA2P compared to TPAP (Figures S5 and S6), suggesting enhanced charge redistribution capability in TPA2P due to its electronic structure.

To benchmark electronic properties, we calculated the ESP and HOMO levels of state-of-the-art carbazole-based SAMs (Figure S7). The dipole moments of Me-4PACz (1.87 D) and 4PADCB (2.47 D) are substantially lower than that of TPA2P (4.87 D), while their HOMO levels (−5.54 eV for Me-4PACz, −5.41 eV for 4PADCB) are shallower than that of TPA2P (−5.66 eV), highlighting the dual advantages of TPA2P in interfacial dipole strength and energy alignment.

To further validate the energy band characteristics, ultraviolet photoelectron spectroscopy (UPS) measurements were employed. As shown in Figures 1d and S8, the work function (WF) is 5.22 eV for ITO/TPA2P and 5.15 eV for ITO/TPA2P. the experimental HOMO are determined to be – 5.47 eV for TPA2P, significantly lower than that of TPAP (−5.29 eV). Although the HOMO energy levels calculated by DFT and measured by UPS differ due to the underlying principles and measurement methods, the overall trends remain consistent. This deeper HOMO level significantly reduces the energy offset with the up layer perovskite film, effectively mitigating *V*_OC_ losses.^22^

Complementary Kelvin probe force microscopy (KPFM) analyses provide further insights into the interfacial electronic properties of the SAM-modified ITO substrates. As shown in Figures 1e and S9, the ITO/TPA2P film exhibits a uniform surface potential distribution that is significantly lower than that of the ITO/TPAP film, indicating a deepening of the WF. Furthermore, the ITO/TPA2P film exhibits a more homogeneous surface potential distribution compared to the ITO/TPAP film, as evidenced by the narrow full width at half-maximum (FWHM) of the surface potential distribution (Figure 1e). We attribute this improved homogeneity to the rigid backbone structure of TPA2P, which enforces molecular alignment and suppresses the formation of distinct crystalline domains—a key source of local variations in surface potential observed in SAMs with flexible spacers.^23^ To directly obtain structural evidence to clarify the crystallinity of TPAP and TPA2P SAM layers, we performed XRD tests (Figure S10). Note that to make the comparison of the results more apparent, we increased the solution concentration and reduced the spin-coating speed for both ITO/TPAP and ITO/TPA2P films (5 mg/mL, 500 rpm). It could be seen that a peak appears at 8.97° for the TPAP film, while no crystallization peak is detected for the TPA2P film, thereby demonstrating that the TPAP film is more prone to crystallization than the TPA2P film. The homogeneity in surface potential could also reduce statistical variations in the *V*_*OC*_ of solar cells, a point that will be discussed in greater detail later. Overall, these results are consistent with the WF and HOMO level measurement from the UPS study, indicating that TPA2P may offer improved interfacial energetic alignment with the perovskite film to facilitate more efficient hole extraction, thereby enhancing device performance.

### Adsorption Properties of SAM Molecules and Buried Interface Modification

The bisphosphonic acid anchoring groups in TPA2P not only modulate its dipole moment and HOMO energy level but also enhance interfacial binding and stability on ITO substrates. To validate this, we conducted molecular dynamics (MD) simulations to track the adsorption of TPAP and TPA2P molecules on ITO during a simulated annealing process (Supplementary Note 2, Figures S11 and S12). After annealing, 9 out of the 10 TPA2P molecules used for the simulation remained anchored to the ITO surface, compared to only 6 TPAP molecules, demonstrating the superior adsorption properties of TPA2P.

Structural analysis reveals that the conjugated ethylene linker in TPA2P imposes steric constraints, fixing the dihedral angle of the double bond at 32.01° (Figure S13a). This rigid backbone likely restricts molecular flexibility, which may influence the arrangement of molecules on the substrate. The resulting interfacial properties are supported by the homogeneous surface potential observed in KPFM measurements, suggesting a uniform molecular distribution.

DFT calculations further corroborate these findings, showing that TPA2P exhibits a substantially higher adsorption affinity on ITO (−15.8 eV) compared to TPAP of −9.4 eV (Supplementary Note 1, Figures 2a and S13). This enhanced absorption is attributed to the bidentate chelation of bisphosphonic acid groups with ITO surface atoms, which promotes robust interfacial bonding. Such strong binding is expected to improve the stability of the TPA2P layer by reducing the desorption and displacement of TPA2P molecules under external factors.

**Figure 2:**
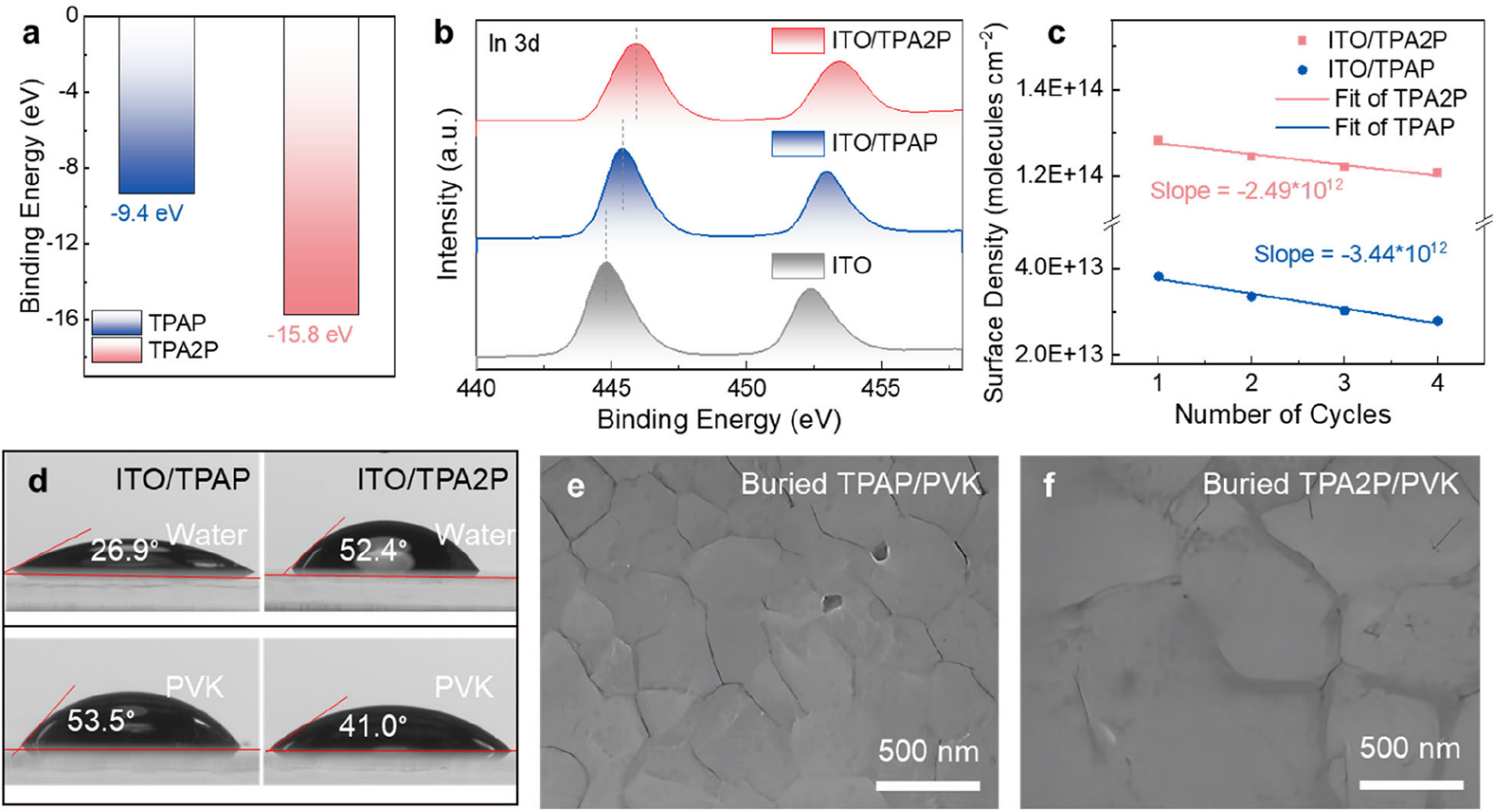
(a) DFT calculation of the binding energy of TPAP and TPA2P on ITO surface. (b) XPS spectra of In 3d core level of bare ITO substrates and ITO substrates covered by TPAP and TPA2P. (c) The surface density variation with the number of cycles. Traverse a test at different scan rates is called one cycle. (d) The contact angle of water (the first line) and perovskite precursor solution (the second line) droplet formed on TPAP and TPA2P films. (e,f) SEM images of the buried perovskite films deposited on (e) TPAP and (f) TPA2P substrates, respectively.

The interfacial interactions between SAMs and ITO were characterized using Fourier-transform infrared (FTIR) spectroscopy and X-ray photoelectron spectroscopy (XPS). As shown in Figures S14 and S15, FTIR spectra reveal distinct shifts in the P–OH vibrational modes for both TPAP and TPA2P, confirming covalent bonding between phosphonic acid groups and ITO surface hydroxyls.^24^ XPS analyses further support this conclusion. Compared to pristine ITO, the In-3d peaks of ITO/TPAP and ITO/TPA2P shift to higher binding energies (444.80–445.39 eV for TPAP and to 445.86 eV for TPA2P, Figure 2b), indicating electron donation from ITO to the SAMs. Additionally, the P 2p peak of ITO/TPA2P (133.94 eV) appears at a lower binding energy than that of ITO/TPAP (134.19 eV), reflecting stronger electron withdrawal by the bisphosphonic acid groups in TPA2P (Figure S16a). These findings are further corroborated by charge density difference analysis (Figure S17).

Deconvolution of O 1s spectra (Figure S16b) identifies three components: lattice oxygen (O_lat_), oxygen vacancies (O_V_), and In–O–P bonds (O–P).^25^ Notably, the O_V_ content decreases from 42.4% (ITO/TPAP) to 25.5% (ITO/TPA2P, Table S1), demonstrating the ability of bisphosphonic acid groups to passivate ITO defects. Quantitative surface analysis (Table S2) reveals a 1.8-fold higher P/In atomic ratio for ITO/TPA2P (0.48) compared to ITO/TPAP (0.26), highlighting the multidentate binding superiority of TPA2P (Table S2). Furthermore, SAM coverage factors derived from C 1s/In 3d peak area ratios (Table S3) show that TPA2P achieves 8 times greater surface coverage (0.24) than TPAP (0.03), underscoring the enhanced interfacial binding of TPA2P.^26^

To evaluate the surface density and binding stability of the SAMs, we performed cyclic voltammetry (CV) measurements using SAM modified ITO substrates as the working electrode across scan rates of 0.025–0.5 V/s (Supplementary Note 3).^27^ The surface density of ITO/TPA2P was estimated to be 1.96 × 10^14^ molecules cm^–2^, significantly higher than that of ITO/TPAP (4.63 × 10^13^ molecules cm^–2^), demonstrating the improved coverage of TPA2P molecules on the substrate (Figures S18–S20). To assess adsorption stability, four consecutive CV cycles were performed, with each cycle comprising a scan rate sweep from 0.5/0.3 to 0.025 V/s (Figures S19 and S20). The corresponding peak current versus scan rate plots (Figures S21 and 2c) reveal a 38% lower desorption rate for ITO/TPA2P (−2.49 × 10^12^ molecules·cm^–2^·cycle^–1^) compared to ITO/TPAP (−3.44 × 10^12^ molecules·cm^–2^·cycle^–1^), clearly indicating the reinforced interfacial anchoring enabled by the bisphosphonic acid groups of TPA2P.

Contact angle measurements provide further insights into the surface properties of SAM-modified ITO (Figure 2d). Interestingly, ITO/TPA2P exhibits a larger contact angle compared to ITO/TPAP when measured with water, indicating a more hydrophobic surface due to the higher coverage of TPA2P. This result aligns well with our surface coverage analysis, which shows significantly greater SAM density for TPA2P. However, when the contact angle is measured using the perovskite precursor solution, the trend reverses: ITO/TPA2P shows a reduced contact angle compared to ITO/TPAP. We attribute this behavior to the bisphosphonic acid linkage in TPA2P, which provides more opportunities for the solvent (DMSO, DMF) or perovskite solutes in the precursor solution to interact with the P=O groups of the bonded SAM. This interaction enhances the spreading of the precursor solution, which is critical for the formation of a uniform perovskite film.^19^

The improved wettability of the precursor solution on ITO/TPA2P is reflected in the crystallinity and morphology of the resulting perovskite layers. To study the morphology of the buried interface, the perovskite films were carefully detached using glass sheet with ultraviolet curing glue, exposing the side of the perovskite layer that was in contact with the ITO/SAM substrate for scanning electron microscopy (SEM) analysis. This approach allowed us to directly examine the interfacial structure and its impact on perovskite crystallization. SEM images reveal distinct differences in the buried interface morphology. As shown in Figures 2e,f and S22, TPA2P-modified perovskite films exhibit smoother buried interface with larger Gaussian fitting average grain sizes 945 nm for ITO/TPA2P and 450 nm for ITO/TPAP-based substrates, facilitating efficient charge transfer at the TPA2P/perovskite interface.^28^ Moreover, photoluminescence (PL) mapping over a 50 × 50 μm^2^ area demonstrates stronger and more uniform emission for TPA2P-based devices (Figures S23 and S24). We attribute these improvements to the rigid backbone structure of TAP2P, which likely interacts with the perovskite precursor components, modulating the perovskite crystallization kinetics and suppressing the nonradiative recombination at the buried interface.

### Morphology and Photoelectric Performance of Perovskite Films

The morphology and crystallinity of the perovskite films were examined with SEM and XRD, respectively. Perovskite films deposited on both TPAP- and TPA2P-modified substrates show pinhole-free surfaces (Figure 3a,b), with TPA2P-based samples exhibiting larger average grain sizes (533 nm vs 402 nm for TPAP, Figure 3c), indicating that TPA2P improves grain growth during perovskite formation. This enhanced grain size is further supported by the cross-sectional data of the ITO/TPA2P/PVK film (Figure S25) and X-ray diffraction (XRD) study. As shown in Figure 3d, the diffraction peak of the TPA2P/PVK film shows higher intensity than TPAP/PVK, which is consistent with the SEM results and suggests higher crystallinity and improved film quality in the TPA2P-modified film. Additionally, atomic force microscopy (AFM) analysis of the perovskite films reveals that TPA2P/PVK sample has a smoother polycrystalline morphology with a lower roughness (Rq = 22.0 nm) compared to the TPAP/PVK sample (Rq = 34.2 nm, Figure S26). PL mapping over 50 × 50 μm^2^ areas shows brighter and more homogeneous emission for TPA2P/PVK (Figures 3e,f and S27), further corroborating its superior crystalline quality.

**Figure 3:**
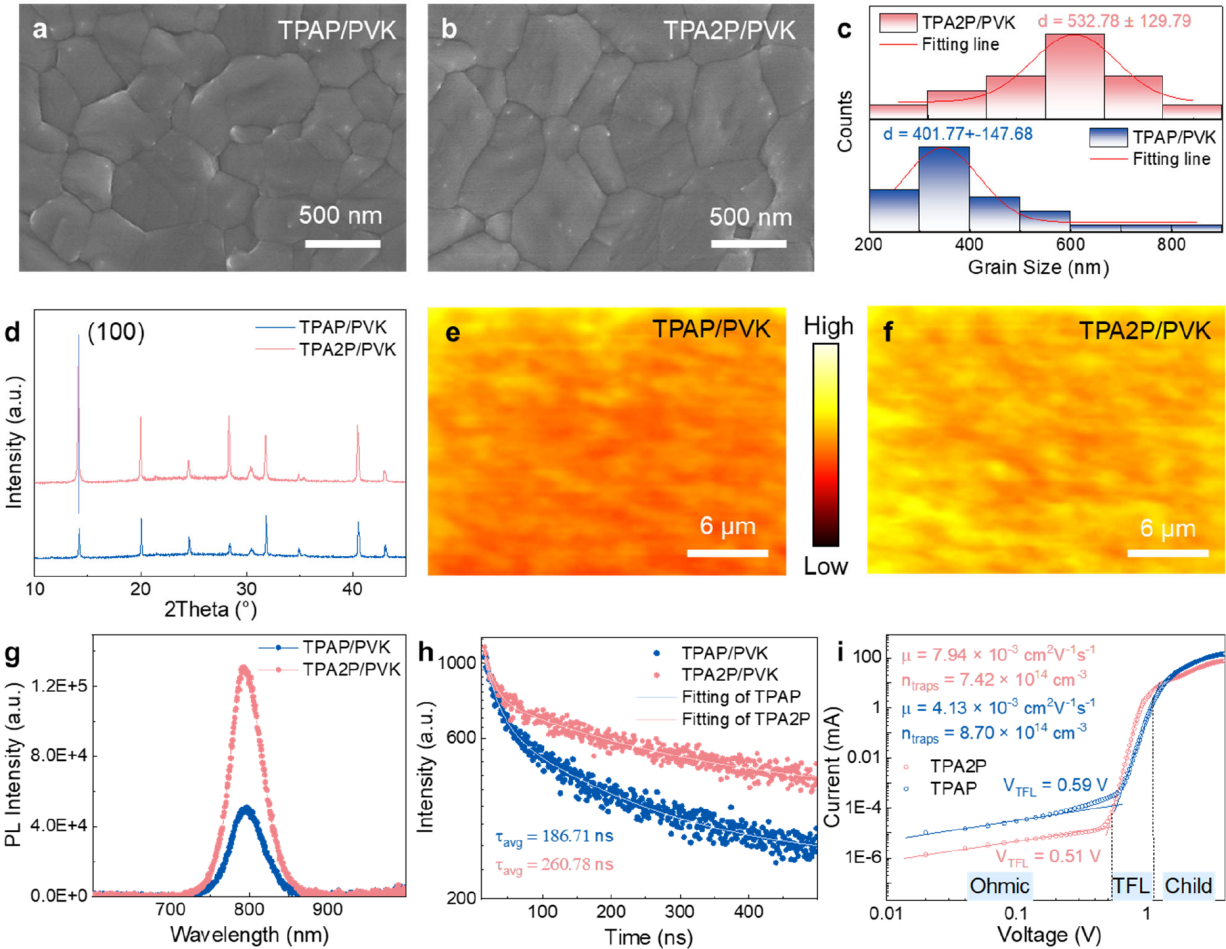
(a,b) SEM images of top surface of perovskite film deposited on (a) TPAP and (b) TPA2P films, respectively. (c) Related grain size statistical distributions of the perovskite films. (d) XRD spectra of different fresh perovskite films. (e,f) PL mapping of the fresh perovskite films deposited on (e) TPAP and (f) TPA2P films, respectively. (g) PL and (h) TRPL spectra of perovskite films deposited on ITO/TPAP and ITO/TPA2P. All films were excited from back side (glass side). (i) Dark *J–V* curves for the hole-only devices with an ITO/SAM (TPAP or TPA2P)/perovskite/PTAA/Ag structure.

To evaluate the optoelectronic properties of the SAM-modified perovskite films, steady-state PL spectra and time-resolved PL (TRPL) decays were analyzed (Supplementary Note 4 and Figure 3g,h). The TPA2P/PVK film exhibits higher PL intensity and a longer carrier lifetime (τ_2_ = 260.78 ns) compared to the TPAP/PVK film (τ_2_ = 186.71 ns, Table S4), The extended τ_2_ is primarily attributed to suppressed interfacial nonradiative recombination losses, consistent with the PL mapping results (Figure 3e,f). To further quantify interfacial nonradiative recombination losses, photoluminescence quantum yield (PLQY) was measured to estimate the quasi-Fermi level splitting (QFLS) of the perovskite films, providing insights into their potential *V*_OC_ losses.^1,29,30^ The TPA2P/PVK film achieves a higher PLQY value (7%) compared to the TPAP/PVK film (4%) (Supplementary Note 5, Figure S28 and Table S5), indicating more efficient radiative recombination of charge carriers. Additionally, the QFLS value for TPA2P/PVK is determined to be 1.21 eV, corresponding to a smaller *V*_OC_ loss of 59.5 mV, in contrast to the 83.2 mV of TPAP/PVK. These results suggest that TPA2P/PVK films possess superior interfacial properties and reduced energy losses.

Furthermore, space charge-limited current (SCLC) measurements were conducted to evaluate the charge transport properties and trap densities of the SAM-modified perovskite films (Supplementary Note 6 and Figure 3i). The TPA2P-based hole-only device exhibits a slightly lower trap density (7.42 × 10^14^ cm^–3^) compared to the TPAP-based device (8.70 × 10^14^ cm^–3^). Moreover, the TPA2P-based device demonstrates enhanced hole transport capabilities, with a hole mobility of 7.94 × 10^–3^ cm^2^ V^–1^ s^–1^, nearly double that of the TPAP-based device (4.13 × 10^–3^ cm^2^ V^–1^ s^–1^). This improved hole mobility, coupled with improved defect passivation, clearly establishes TPA2P as a high-performance HTM for inverted PSCs.

### Device Performance

We fabricated perovskite solar cells with an inverted structure of ITO/SAM (TPA2P or TPAP)/Cs_0_._0__5_FA_0_._9__5_PbI_3_/C_60_/BCP/Ag (Figure 4a) to evaluate the photovoltaic performance of TPA2P as a HTL, with TPAP serving as a reference material for comparison. The best-performing TPAP-based device achieved a PCE of 23.88%, with a *V*_OC_ of 1.17 V, a *J*_SC_ of 25.09 mA cm^–2^, and a FF of 81.58% (Figures 4b and S29). In contrast, devices incorporating TPA2P exhibited a markedly improved PCE of 26.11%, accompanied by a stabilized power output (SPO) of 26.08–25.61% after 400 s (from 23.73 to 21.41% for the TPAP device, Figure S30). The optimized TPA2P device demonstrated a *V*_OC_ of 1.18 V, a *J*_SC_ of 25.99 mA cm^–2^, and an FF of 85.03%. The *J*_SC_ value aligns closely with the integrated current density (25.24 mA cm^–2^) derived from external quantum efficiency (EQE) measurements (Figure 4c), showing a discrepancy of <3% relative to the *J–V* data. The optical bandgap of the perovskite layer, determined from the first derivative of the EQE spectrum, was calculated to be 1.54 eV (Figure S31). The improvement in these key parameters is mainly attributed to the more compatible HOMO energy level between TPA2P and the perovskite, which can effectively reduce *V*_OC_ loss and facilitate charge extraction. Additionally, TPA2P has been shown to significantly enhance the crystallinity of the perovskite film, thereby reducing interface defect states, which also contributes to the increase in *J*_*SC*_ and FF.

**Figure 4:**
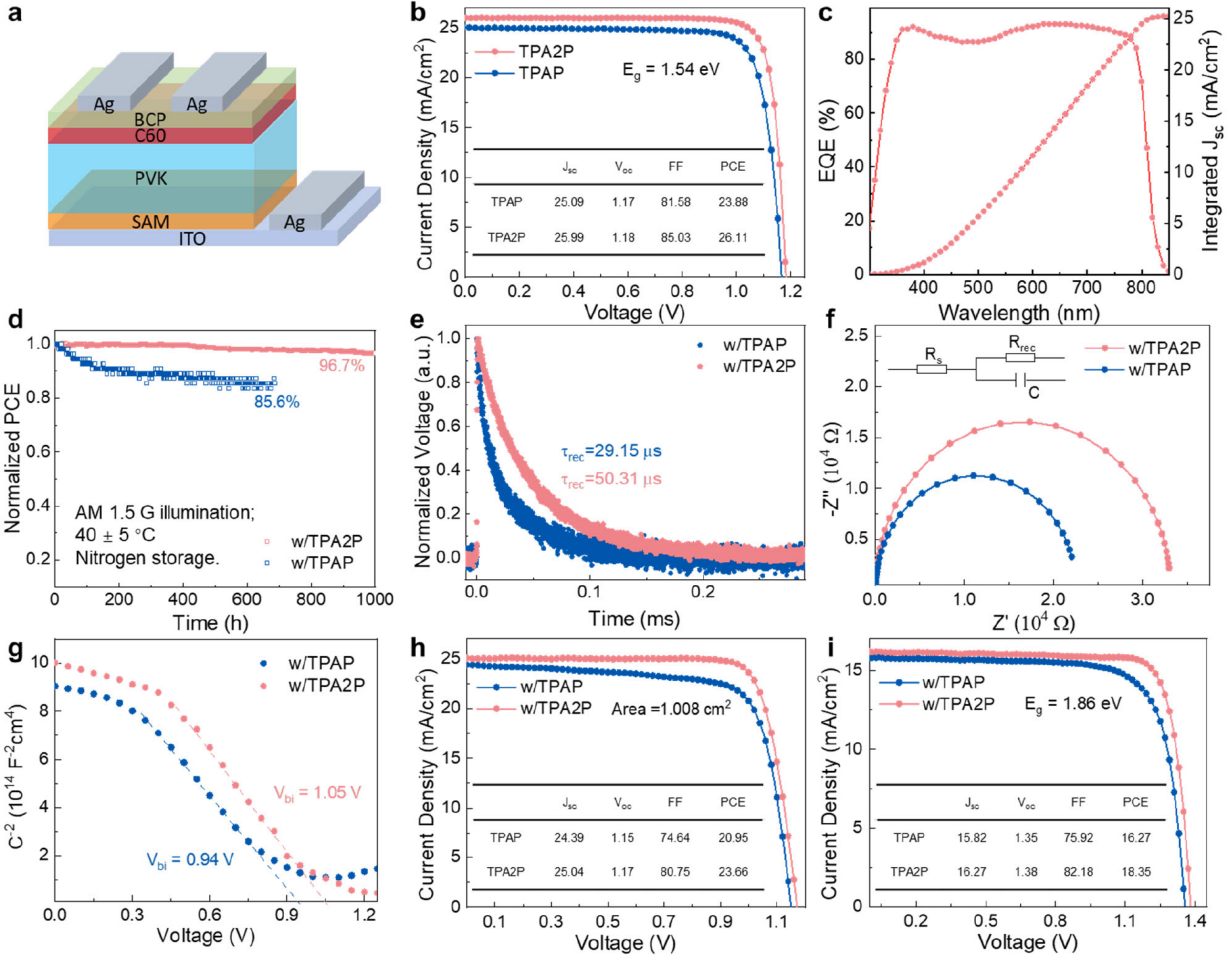
(a) Device structure of PSC. (b) Optimized *J–V* curves of the TPAP- and TPA2P-based devices. (c) EQE spectra and integrated current densities of TPA2P-based devices. (d) MPP tracking under N_2_ and simulated 1 sun AM 1.5 G illumination for uncooled devices (reaching an operating temperature of 40 ± 5 °C). (e) TPV decay for the TPAP- and TPA2P-based devices. (f) Nyquist plots and (g) Mott–Schottky plots of the TPAP- and TPA2P-based devices. (h) *J–V* curves of the large area (1.008 cm^2^) TPAP- and TPA2P-based devices. (i) *J–V* curves of the wide bandgap (1.86 eV) devices based on TPAP and TPA2P.

To contextualize these results, we also benchmarked TPA2P against the widely studied carbazole-based SAM material (4-(7H-dibenzo­[c,g]­carbazol-7-yl)­butyl) phosphonic acid (4PADCB, Figure S32).^4,28,29^ The champion 4PADCB-modified device delivered a PCE of 23.97%, with a *V*_OC_ of 1.165 V, a *J*_SC_ of 24.46 mA cm^–2^, and an FF of 84.12%, underscoring the superior performance of TPA2P in all key photovoltaic parameters. Long-term stability tests under accelerated aging conditions (maximum power point (MPP) tracking, 40 ± 5 °C, AM 1.5G illumination, N_2_ atmosphere) highlighted the robustness of TPA2P-based devices. Unencapsulated TPA2P devices retained 96.7% of their initial PCE after 1000 h, whereas TPAP-based counterparts degraded to 85.6% of their initial performance (Figure 4d). This stark contrast in stability underscores the critical role of conjugated bisphosphonic acid structure of TPA2P in mitigating interfacial degradation. Collectively, these results demonstrate that TPA2P not only elevates device efficiency beyond state-of-the-art SAM-HTLs but also addresses persistent challenges in operational stability, positioning it as a promising HTL for high-performance inverted PSCs.

To further elucidate the charge carrier dynamics of the PSCs, we conducted transient photovoltage (TPV) and transient photocurrent (TPC) measurements (Supplementary Note 7, Figures 4e and S33). The TPA2P-based device exhibits a longer charge recombination lifetime of 50.31 μs, compared to 29.15 μs for the TPAP-based device, demonstrating its enhanced defect passivation and suppression of nonradiative recombination.^31^ TPC measurements further revealed a shorter charge transfer time of 1.48 μs for the TPA2P-based device, compared to 2.69 μs for the TPAP-based device, highlighting its faster and more efficient charge transport and extraction capability. These results collectively underscore the dual capability of TPA2P in reducing recombination losses while promoting efficient charge extraction.

We also analyzed the light intensity dependence of *V*_OC_ to quantify defect-assisted charge recombination (Supplementary Note 8 and Figure S34). The TPA2P-based device exhibited a near-ideal slope of 1.03*kT*/*q*, significantly lower than the 1.24*kT*/*q* observed for TPAP-based devices, indicating suppressed trap-assisted Shockley–Read–Hall (SRH) recombination.^32,33^ Additionally, the power-law exponent (α) derived from the *J*_SC_-light intensity relationship reflects the recombination mechanism. The TPA2P-based device achieved an α value of 0.873 (vs 0.868 for TPAP), approaching the ideal value of 1, which suggests minimal space-charge effects and reduced bimolecular recombination while maintaining efficient charge collection.^34^

Impedance spectroscopy further corroborated the superior charge transport properties of TPA2P-based devices. Measurements revealed a significant lower series resistance (*R*_s_ = 5.1 Ω for TPA2P-based devices and 25.3 Ω for TPAP-based devices) and higher recombination resistance (*R*_res_ = 3312.0 Ω for TPA2P-based devices and 2251.0 Ω for TPAP-based devices) (Figure 4f). These improved metrics correlate with the observed enhanced *V*_OC_ and FF, demonstrating how the molecular design of TPA2P reduces nonradiative losses.

The origin of the reduced voltage deficit of TPA2P-based devices was further elucidated through Mott–Schottky analysis, which revealed an enhanced built-in potential (*V*_bi_ = 1.05 V vs 0.94 V for TPAP-based devices; Figure 4g). This increase in *V*_bi_ strengthens the internal electric field at the HTL/perovskite interface, synergistically improving charge separation while suppressing interfacial recombination.^35^ The pronounced distinctions in built-in electric fields do not directly translate to corresponding *V*_OC_ variations, suggesting differences in nonradiative recombination losses, interfacial quality, and charge extraction dynamics between the two devices.

Finally, capitalizing on the inherent advantages offered by TPA2P, we moved forward to demonstrate the area scalability of TPA2P by fabricating larger area devices (1.008 cm^2^) and evaluating its compatibility with different perovskite compositions using wide-bandgap devices (1.86 eV, Cs_0.2_FA_0.8_PbI_1.5_Br_1.5_). The TPA2P-based large-area device delivers a PCE of 23.66%, representing a 13% improvement over its TPAP-based counterpart (20.95%, Figures 4h and S35). Likewise, the TPA2P-based wide bandgap devices achieve a PCE of 18.35%, with all three photovoltaic parameters improved simultaneously, resulting in an overall PCE enhancement of over 10% compared to the TPAP-based counterparts (Figures 4i). In addition to TPAP, the properties and performance of widely studied SAMs were compared with those of our TPA2P (Table S6). These findings highlight conjugated bisphosphonic acid engineering as a universal strategy for achieving high-efficiency, scalable perovskite photovoltaics.

## Conclusions

In this work, we demonstrate that conjugated bisphosphonic acid anchoring groups significantly enhance the binding strength and stability of TPA2P-based SAMs on ITO substrates. The incorporation of a conjugated double-bond linker facilitates efficient electron delocalization from the TPA core to the electron-withdrawing bisphosphonic acid groups, effectively lowering the HOMO level and increasing the molecular dipole moment. This electronic tuning reduces *V*_OC_ losses and enhances charge extraction efficiency. Moreover, the optimized molecular structure of TPA2P promotes uniform SAM formation, which contributes to improved interfacial contact and enhanced perovskite film quality. As a result, TPA2P-based inverted perovskite solar cells achieve an impressive PCE of over 26%, along with enhanced operational stability. Given the widespread reliance on monophosphonic acid anchors in SAM design, our bisphosphonic acid strategy offers a broadly applicable approach for advancing high-performance optoelectronic devices, paving the way for more efficient and stable perovskite photovoltaics.

## Supplementary Material



## Notes

Materials and methods; experimental details; additional simulation details and characterization data, including NMR, ESP, UV–vis, HOMO, LUMO, KPFM, molecule dynamics, FTIR, XPS, charge density difference, CV, PL mapping, PLQY, QFLS, *J–V*, SPO, EQE, TPC, light intensity dependence of *V*_OC_; and stability tests and photograph of large area device (PDF)
