# Supplementary material for: Conjugated Bisphosphonic Acid Self-Assembled Monolayers for Efficient and Stable Inverted Perovskite Solar Cells
Source: J Am Chem Soc. 2025 Jun 30;147(28):24662–71. doi: 10.1021/jacs.5c05801 (PMC12272685; doi:10.1021/jacs.5c05801)
Supplement: Supplementary file 1 [file ja5c05801_si_001.pdf]

Supporting Information

Conjugated Bisphosphonic Acid Self-Assembled Monolayers for Efficient and Stable Inverted Perovskite Solar Cells

Songyang Yuan[a,b,‡], Chengda Ge[b,‡], Tianyi Zhang[a,‡], Gengyang Su[c], Quanrun Qiu[b], Guanhua Ren[b], Lingyi Ke[b], Gengxin Du[b], Guangruixing Zou[b], Nan Zhang[b], Hui Liu[b], Qingduan Li[a], Tao Jia[a,d,], Yue-Peng Cai [a], Shengjian Liu[a,*] and Hin-Lap Yip[b,e,f,g,*]*

[a]School of Chemistry, Guangzhou Key Laboratory of Materials for Energy Conversion and Storage, Key Laboratory of Electronic Chemicals for Integrated Circuit Packaging, South China Normal University (SCNU), Guangzhou 510006, China

[b]Department of Materials Science and Engineering, City University of Hong Kong, Kowloon, Hong Kong 999077, China

[c]Guangdong Zenithnano New material co., Ltd. Guangzhou 510006, China

[d]School of Optoelectronic Engineering, School of Mechanical Engineering, Guangdong Polytechnic Normal University, Guangzhou, 510665, China

[e]Hong Kong Institute for Clean Energy (HKICE), City University of Hong Kong, Kowloon, Hong Kong 999077, China

[f]State Key Laboratory of Marine Pollution, City University of Hong Kong, Kowloon, Hong Kong 999077, China

[g]School of Energy and Environmental Science, City University of Hong Kong, Kowloon, Hong Kong 999077, China

[*]Corresponding authors. Email: tjia@gpnu.edu.cn (Tao Jia).; shengjian.liu@m.scnu.edu.cn (Shengjian Liu); a.yip@cityu.edu.hk (Hin-Lap Yip);

[‡]These authors contributed equally to this work.

1. Materials and Methods

Materials:

4-(diphenylamino)benzaldehyde (TPA-CHO) and tetraethyl methylenebis(phosphonate) were purchased from Bide Pharmatech Ltd. All chemical reagents were employed as received without further purification. N,N-dimethylformamide (DMF, 99.99%), dimethyl sulfoxide (DMSO, 99.70%), isopropanol (IPA, 99.50%), and chlorobenzene (CB, 99.90%) were procured from J&K Scientific. Metal halide precursors including cesium iodide (CsI), methylammonium iodide (MAI), methylammonium chloride (MACl), lead (II) chloride ($PbCl_2$), lead bromide ($PbBr_2$, 99.9%) and ethylenediammonium diiodide ($EDAI_2$) were obtained from Xi'an Polymer Light Technology Corp. High-purity lead iodide (PbI_2, 99.9985%), and Me-4PACZ were supplied by Tokyo Chemical Industry Co., Ltd. (TCI). Formamidinium iodide (FAI) was sourced from Dysol Co., Ltd.

Materials characterization:

The ^{1}H NMR and ^{13}C NMR spectra were measured on a Bruker AVANCE NEO (600 MHz) spectrometer with tetramethylsilane (TMS) as the internal reference at room temperature.

General Experimental Details

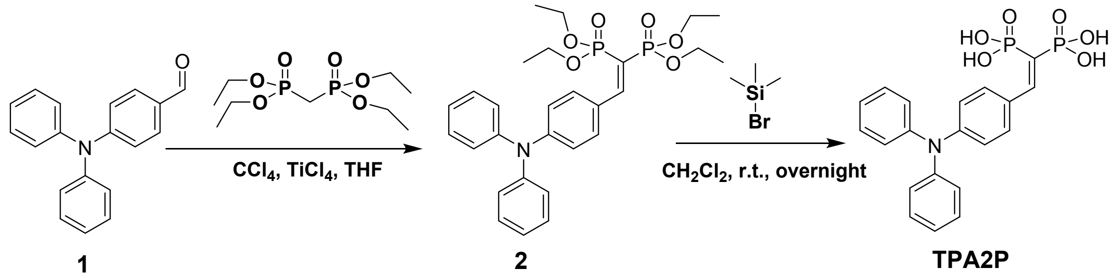

Scheme S1. The detailed synthetic routes of the SAM TPA2P.

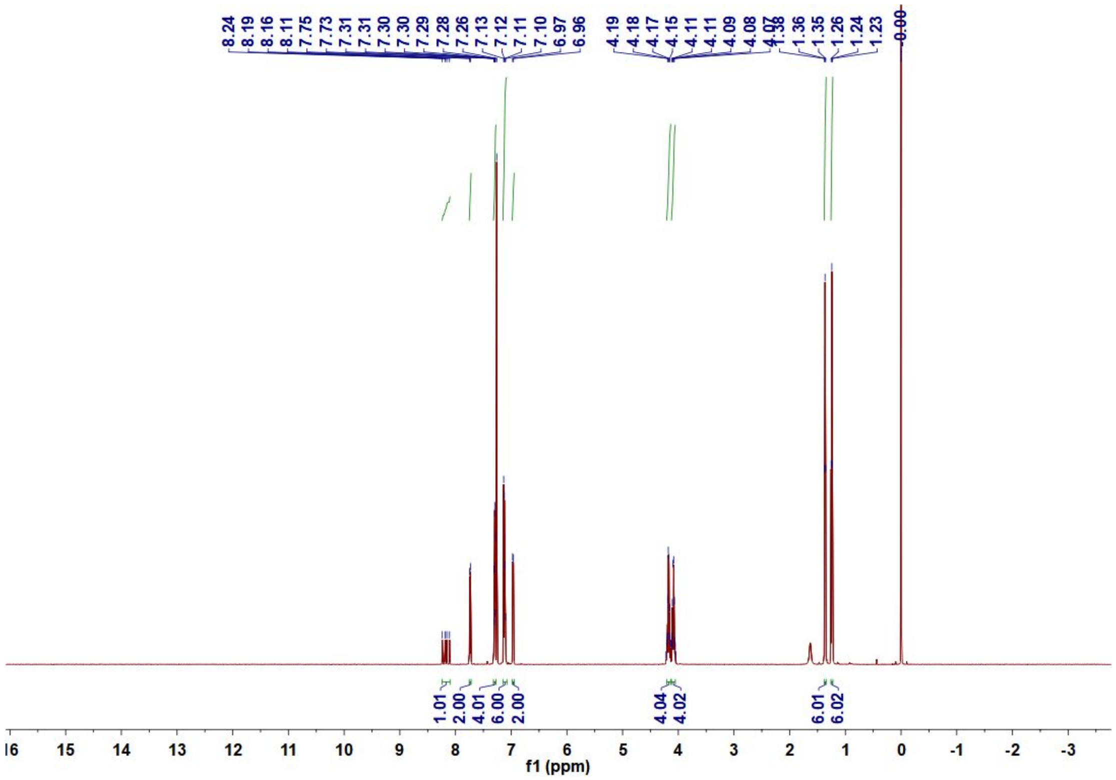

Figure S1. The ^{1}H NMR spectra of compound 2.

Tetraethyl (2-(4-(diphenylamino)phenyl)ethene-1,1-diyl)bis(phosphonate) (Compound 2):

Tetraethyl (2-(4-(diphenylamino)phenyl)ethene-1,1-diyl)bis(phosphonate): Titanium tetrachloride($TiCl_4$, 5 mL) was added into carbon tetrachloride (CCl_4, 15 mL) under ice bath and Ar atmosphere. Tetrahydrofuran(30mL) was added dropwise into the carbon tetrachloride solution. The 4-(diphenylamino)benzaldehyde (3.28 g, 12 mmol) and tetraethyl methylenebis(phosphonate) (6 g, 20 mmol) were dissolved in 30mL tetrahydrofuran formulated the mixture of reactants. The reactants mixture was added into the carbon tetrachloride solution. 4-Methylmorpholine (1 mL) was added into the solution. The mixture was further stirred to the room temperature, and then evaporated the solution. The solid was tetraethyl (2-(4-(diphenylamino)phenyl)ethene-1,1-diyl)bis(phosphonate). ^{1}H NMR (600 MHz, $CDCl_3$) δ 8.17 (dd, J = 48.0, 29.5 Hz, 1H), 7.74 (d, J = 8.8 Hz, 2H), 7.32 – 7.27 (m, 4H), 7.12 (dd, J = 13.5, 7.4 Hz, 6H), 6.97 (d, J = 8.9 Hz, 2H), 4.21 – 4.14 (m, 4H), 4.13 – 4.05 (m, 4H), 1.36 (t, J = 7.1 Hz, 6H), 1.24 (t, J = 7.1 Hz, 6H). ^{13}C NMR (151 MHz, $CDCl_3$) δ 161.11, 161.10, 150.58, 146.53, 133.35, 129.54, 125.81, 124.45, 119.85, 62.48, 62.44, 62.33, 62.29, 16.37, 16.33, 16.20, 16.15.

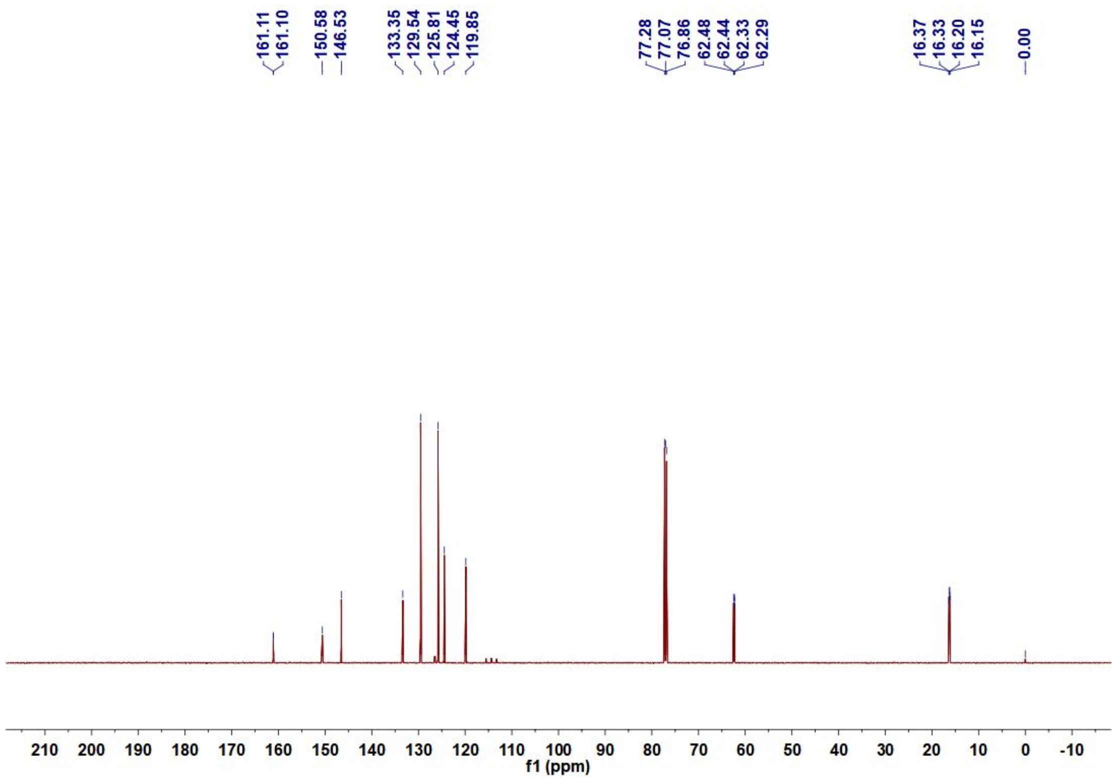

Figure S2. The ^{13}C NMR spectra of compound 2.

(2-(4-(Diphenylamino)phenyl)ethene-1,1-diyl)bis(phosphonic acid) (Compound TPA2P): Tetraethyl (2-(4-(diphenylamino)phenyl)ethene-1,1-diyl)bis(phosphonate) (420 mg, 0.7727 mmol) was added into the dry dichloromethane (10 mL) under Ar atmosphere. The 12 equivalent trimethyl bromide silane (1.351 g, 1.165 mL) was added into the solution and stirred overnight. The methanol (42 mL) and water (0.166 mL) was added to the solution and stirred for an hour. Then the solution was evaporated. The solid was dissolved into a 10 mg/mL solution in MeOH for next-step use.

Perovskite Precursor Preparation

Normal Bandgap:

A 1.4 M perovskite precursor solution with a nominal composition $Cs_{0.05}FA_{0.95}PbI_3$ was prepared by dissolving stoichiometric amounts of FAI, PbI_2, and CsI in a mixed solvent of DMF) and DMSO (volume ratio 4:1). To optimize film formation and crystallinity, the precursor solution was supplemented with excess PbI_2 (5 wt%), $PbCl_2$ (3 wt%), and MACl, (10 wt%), along with 4-fluorophenethylammonium iodide (4F-PEAI, 1.4 mg) in 1 ml precursor solutions as an additive. The resulting solution was stirred at room temperature for 1 hour at least with no subsequent filtration before use.

1.86 eV Wide-Bandgap (WBG):

The 1.86 eV WBG perovskite precursor solution was prepared following a similar procedure, CsI (62.4mg) FAI (165.1 mg) PbI_2 (138.3mg), $PbBr_2$ (330.3 mg), henethylammonium acetate (PEAAc, 2%) dissolved in 1 mL of DMF/DMSO mixed solvent (4:1 v/v). The solution was stirred overnight at room temperature, and no filtration was required before use.

Device Fabrication

Single-junction normal bandgap PSCs:

The pre-patterned ITO glass substrates were subjected to a rigorous cleaning process involving sequential sonication with detergent, deionized water, acetone, IPA, each for 15 minutes. Following this, the cleaned ITO substrates were dried in an oven at 75 °C for 24 hours and treated with oxygen plasma for 30 minutes prior to use. The solutions of SAM materials (TPAP and TPA2P) were prepared by dissolving with IPA to a concentration of 1.0 mg/mL, respectively. This solution was spin-coated onto the ITO substrates at 4,000 rpm for 30 seconds, followed by annealing at 100 °C for 10 minutes. Subsequently, 40 μL of the perovskite precursor was spin-coated at 1,000 rpm for 10 s 4,000 rpm for 50 seconds, with 180 μL of CB antisolvent was introduced at the film 5 seconds before the end of the spin-coating process. This was followed by annealing at 100 °C for 50 minutes. The PI solution (0.3 mg/mL in IPA) was then spin-coated onto the perovskite layer at 3,000 rpm for 30 seconds and annealed at 100 °C for 10 minutes.

All spin-coating processes were conducted in a nitrogen-filled glovebox, maintaining O_2 and H_2O levels below 5 ppm, at a controlled temperature of approximately 20 °C. Finally, 25 nm layer of C_{60}, 6 nm layer of BCP, and 100 nm Ag were thermally evaporated in a high-vacuum chamber (< 2×10^{-6} torr) through a metal shadow mask with an aperture area of 0.04 cm². This was followed by the thermal evaporation of a 100 nm MgF_2 layer onto the glass side of the devices, serving as an antireflection layer.

Single-junction 1.86 eV bandgap PSCs:

The perovskite layers were prepared using a two-step spin-coating process with 1000 rpm for 10 s and a second step 4000 rpm for 40 s. During the spin-coating, 180 μL of chlorobenzene (CB) antisolvent was carefully dripped onto the wetted film 25 s before the process concluded. The film was then annealed at 100 °C for 10 minutes. Subsequently, a passivation layer was applied by spin-coating $EDAI_2$ (0.5 mg/mL in IPA) at 3000 rpm for 30 s, followed by annealing at 100 °C for an additional 10 minutes.

Large area (1 cm^2) normal bandgap PSCs:

Preparation of Perovskite Precursor Solution: The 1.40 M perovskite precursor solution was prepared by mixing PbI_2, CsI, FAI in DMF: DMSO (4:1/v:v) mixed solvent with a chemical formula of $Cs_{0.05}FA_{0.95}PbI_3$. Moreover, 5 mol% of excess PbI2, 10 mol% of MACl and 3mol% of excess $PbCl_2$ were added as additive to improve the device performance.

Large area devices fabrication: The patterned ITO substrates were treated with oxygen plasma for 30 min and transferred into an N_2-filled glove box immediately, The SAM solution was dripped evenly on ITO substrates and spin-coated at 3000 rpm for 30 s and then annealed at 100 °C for 10 min to achieve the deposition of the holt transport layer. After cooling, 80 μL of the prepared perovskite precursor solution was spun on a SAM-based substrate at 1000 rpm for 10 s and 4000 rpm for 60 s. CB (300 μL) was added dropwise onto the center of substrates at 5 s before the end of spin coating program. The as-prepared perovskite films were immediately transferred to a hotplate and annealed at 100 °C for 30 min. For the 2D passivation layer, 70 μL of PDADI solution (1 mg/mL in IPA) was spin-coated onto the perovskite surface at 5000 rpm for 30 s with subsequent annealing treatment at 100 °C for 10 min. Finally, a 25 nm C60,

6 nm BCP and 100 nm Ag were thermally evaporated in a high-vacuum chamber (< 2 × 10^{-6} torr) through a metal shadow mask.

Characterizations

Film characterizations:

Cyclic voltammetry measurement was carried out using electrochemical analyzer CHI760E (Chenhua Co. Ltd, Shanghai, China) at room temperature, employing a conventional three-electrode system. This system consisted of a glassy carbon electrode as the working electrode, a Pt wire as the counter electrode, and an Ag/AgCl (saturated KCl) reference electrode. The dark J–V characteristics of the holeonly devices were measured using a Keithley 2400 SourceMeter. Steady-state photoluminescence (PL), time-resolved PL (TRPL), and photoluminescence quantum yield (PLQY) spectra were measured using an FLS1000 photoluminescence spectrometer system (Edinburgh). Excitation was achieved with 375 nm light for PL and a pulsed 375 nm laser for TRPL. Ultraviolet photo-electron spectroscopy (UPS) and X-ray photoelectron spectroscopy (XPS) analysis was conducted using a Thermo Fisher ESCALAB XI+ spectrometer, utilizing non-monochromatic He I UV light with an energy of 21.21 eV acting as the excitation source at an energy resolution of 50 meV. UV-vis absorption spectra were acquired using an Agilent 8454 spectrophotometer. The morphology of the thin-film samples in top view of the devices were examined using scanning electron microscopy (SEM, QUATTRO S). Powder and thin-film X-ray diffraction (XRD) characterizations were conducted using a D2 Phaser instrument with Cu Kα radiation (wavelength of 1.5418 Å). ^{1}H NMR and ^{13}C NMR NMR spectra were measured on a Bruker AVANCE NEO (600 MHz) or Bruker AvanceⅢ HD (500MHz) spectrometer with tetramethylsilane (TMS) as the internal reference at room temperature or 135 ^{o}C (high temperature NMR test). Fourier-transform infrared (FTIR) spectroscopy was conducted by Fourier transform infrared spectrometer (Tensor 27, Germany Bruker). FTIR spectra were taken from KBr pellet-pressed power samples to amplify the signal. Atomic force microscope (AFM) and Kelvin probe force microscopy (KPFM) data were acquired via Bruker Dimension Kelvin probe force microscopy in Potential Channel equipped with

PFQNE-AL probe. PL mapping was acquired using a laser scanning confocal Raman spectrometer (LabRAM HR Evolution) with excitation at 405 nm on the perovskite thin film. The contact angle measurements were performed using a drop shape analyzer (DSA 25S, Kruss).

Device characterizations:

The current density-voltage (J-V) characteristics of the devices were measured in a nitrogen-filled glovebox using Keithley 2400 Source Meter, under simulated sunlight (SS-F5, EnliTech). To achieve an AM 1.5G (100 mW/cm^2) solar simulator light intensity, a National Renewable Energy Laboratory (NREL) calibrated silicon solar cell with a KG-2 filter was used. During the J-V testing, perovskite solar cells were covered with a shading mask with an aperture area of 0.04 cm^2 to ensure the accuracy of the current density derived from the J-V curves. The J-V measurements were performed in sweep mode with both reverse and forward scans at a scan rate of 10 mV/s and a step size of 0.02 V. Additionally, external quantum efficiency (EQE) curves were obtained using an EQE measurement system (QE-R, EnliTech). Electrical impedance spectroscopy (EIS) measurement was carried out using electrochemical analyzer CHI760E (Chenhua Co. Ltd, Shanghai, China) at room temperature. The perovskite solar cells (PSCs) were operated at their maximum power point (MPP) while being illuminated by an LED source covering wavelengths from 400 to 1,000 nm. Throughout the test, the sample chamber was maintained in a nitrogen-filled glovebox. The TPV technique using PAIOS was based on monitoring the photovoltage decay upon a small optical perturbation during different constant bias light-intensity. TPC measurement was performed with a LED light illumination and a small perturbation 500 nm laser pulse (pulse width of ~7 ns). The laser is supplied by a commercial Nd:YAG OPO (Oppolette), at a constant repetition rate of 20 Hz. The photocurrent is generated with a photovoltage signal coupled to a 50 Ω resistor, recorded by an oscilloscope (Tektronix TDS 3052C).

2. Supplementary Notes

Supplementary Note 1. First-principles calculations.

The values of the binding energies are calculated by density functional theory (DFT) method with the software of CP2K v2024.1 program utilizing a DZVP-MOLOPT-SR-GTH basis and the PBE exchange-correlation functional.[1-2] The energy cut-off for the electron density expansion is 350 Ry, and the convergence threshold of density matrix of inner self-consistent field (SCF) is 10^{-6} eV and 0.05 eV $Å^{-1}$ for the norm of the atomic forces. For the binding energy calculation, the binding energy $(\Delta E_{binding})$ is defined as:

$$\Delta E_{binding} = E_{slab+molecule} - E_{slab} - E_{molecule} \quad (1)$$

Here, E_{slab} and $E_{molecule}$ are the total energy of the slab and adsorbed SAM molecule, respectively.

The charge density differential analysis is performed to elucidate the charge redistribution characteristics during the interfacial interaction. The charge density differential (Δρ) is calculated using the formula:

$$\Delta\rho = \rho_{slab+molecule} - \rho_{slab} - \rho_{molecule} \quad (2)$$

where $\rho_{slab+molecule}$ represents the electron density of the optimized system, ρ_{slab} and $\rho_{molecule}$ are the electron density of ITO substrate and adsorbed SAM molecule in the optimized system. To ensure computational consistency, all atomic positions in the isolated state maintained identical coordinates to those in the optimized configuration. The charge density distributions are visualized through isosurface analysis with a threshold value of 0.02 e/$Å^3$.

The ELF is systematically computed to characterize the spatial distribution of localized electrons and bonding interactions within the system. All calculations were performed within the framework of DFT calculation, with wavefunction data extracted from SCF converged charge densities. The ELF values, ranging from 0 to 1, were visualized using the Multiwfn package.[3-4] Basin analysis was further conducted to partition the ELF field into chemically meaningful domains, enabling quantitative

assessment of electron localization volumes and their topological connectivity.

The Electrostatic potential (ESP), Highest occupied molecular orbital (HOMO) and Lowest unoccupied molecular orbital (LUMO) of single SAM materials were calculated using ORCA 5.0.1. software.[5] The geometry optimization at the level of theory of B3LYP/def2-SVP. Then, total energies were further calculated at the level of theory of B3LYP/def2-TZVP based on single-point calculations of the optimized structures.

Supplementary Note 2. The molecular dynamics simulations.

Molecular dynamics simulations are carried out to investigate the adsorption distribution of the SAM molecules near the confined ITO substrates from the atomic level. The substrates are set as rigid to ensure that the atoms of the ITO are fixed during the simulation. For the simulation, a geometry optimization is firstly employed to relax the simulation box. Then, a canonical (NVT) ensemble with a 1.0 fs time step is employed to optimized the simulation box, where the temperature is set to 400 K. The temperature is kept via the Nose-Hoover thermostat. The optimization time is set to 5.0 ns, which is long enough to obtain a stable system. In all the MD simulation, the motion of atoms is described by classical Newton ' s equation, which is solved using the velocity-Verlet algorithm.

Supplementary Note 3: Cyclic voltammetry (CV) analysis.

The surface density of SAM molecules onITO could be determined from cyclic voltammetry using the equation below:

$$i_p = \frac{n^2F^2}{4RTN_A}A\Gamma v \quad (3)$$

where i_p is the oxidative peak current (A), v is the voltage scanrate (V/s), n is the number of electrons transferred, T is the temperature (K), the Fara-day constant F = 96485.33 C mol^{-1}, the universal gas constant R = 8.3144 J K mol, the Avogadro constant N = 6.022*10^{23} mol^{-1}, and the electrode surface area A = 1.5 cm^{-2} in this work, and Γ (molecules cm^{-2}) is the surface density that could be obtained by calculating the slope of i_p versus v.

Supplementary Note 4: TRPL analysis.

The quality and passivation effect of perovskite thin films is investigated by time-resolved PL (TRPL) spectroscopy. The TRPL data are fitted using a bi-exponential function:[6-7]

$$y = y_0 + A_1 e^{\left(\frac{-\tau}{\tau_1}\right)} + A_2 e^{\left(\frac{-\tau}{\tau_2}\right)} \quad (4)$$

Where A_1 and A_2 are the correlation coefficient of carrier lifetime τ_1 and τ_2, respectively. y_0 is a fitting constant, τ_1 and τ_2 are the decay components related to a fast bimolecular recombination and a slow trap assisted recombination process, respectively. The average carrier recombination lifetime (τ_{avg}) could be calculated by equation:

$$\tau_{avg} = \frac{\sum A_i \tau_i^2}{\sum A_i \tau_i} \quad (5)$$

A larger τ_{avg} could be index for better quality of perovskite.

Supplementary Note 5: Calculation of the quasi-Fermi level splitting (QFLS) based on the PL quantum yield (PLQY).

To investigate the non-radiative recombination at the interface between the different SAM layers and perovskite, we calculated the QFLS of 1.53 eV bandgap perovskite films on different SAM layers on the ITO substrate by using PLQY measurements. The direct relation between QFLS and PLQY follows the equation:[8]

$$QFLS = QFLS_{rad} + K_B T ln(PLQY) = K_B \mathrm{T} \ln\left(\frac{J_G}{J_{0,rad}} PLQY\right) \quad (6)$$

Here, *QFLS* is the difference between the electron and hole quasi-Fermi levels in the perovskite layer, k_B is the Boltzmann constant, and T is the temperature (300 K). J_G is the generation current density under illumination, which is approximated to the short-circuit current density J_{SC} of devices in this case. $J_{0,rad}$ is the dark radiative recombination saturation current density.

According to the detailed balance theory, the $J_{0,rad}$ could be calculated by the following equations:

$$J_{0,rad} = q\int_0^{\infty} EQE_{PV}(E)\phi_{BB}(E)dE \quad (7)$$

$$\phi_{BB}(E) = \frac{2\pi E^2}{h^3c^2} * \frac{1}{exp\left(\frac{E}{K_BT}\right)-1} \quad (8)$$

Where q is the elementary charge, EQE_{PV} is the photovoltaic external quantum efficiency, E is the photon energy, ϕ_{BB} is the black-body radiative spectrum, h is the Planck constant, and c is the light speed in a vacuum.

External quantum efficiency (EQE) of the PSCs and the emitted spectral photon flux could be calculated when the device is in equilibrium with the black-body radiation (T = 300 K). Based on equations 4 and 5, the $J_{0,rad}$ is calculated similarly as 2.87×10^{-19} Am^{-2} for all systems independent of the bottom charge transport layer. Subsequently, combined with the PLQY value of the samples with the different SAM layers underneath, the QFLS of the samples could be obtained according to equation 3. Besides, the V_{OC} non-radiation recombination loss $\Delta V_{OC}^{non-rad}$ could be obtained according to the following equation:

$$\Delta V_{OC}^{non-rad} = \frac{QFLS_{rad}-QFLS}{q} = \frac{k_BTln(PLQY)}{q} \quad (9)$$

Supplementary Note 6: SCLC analysis.

The dark *J–V* curves of the hole-only device could be divided into three parts: Ohmic region, trap-filling limited region and the trap-free Child region. The trap density (N_t) of perovskite films could be estimated using the following equation:[9]

$$N_t = \frac{2\varepsilon\varepsilon_0 V_{TFL}}{qd^2} \quad (10)$$

where d is the film thickness. q is the elementary charge (1.602×10^{-19} C). ε and ε_0 are the relative dielectric constant of the perovskite and the vacuum permittivity (8.8542×10^{-14} F/cm), respectively. V_{TEL} is the onset voltage of the trap-filled-limit. The dielectric constant ε is estimated from impedance measurement using the equation:[10-11]

$$\varepsilon = \frac{Cd}{A\varepsilon_0} \quad (11)$$

where C is capacitance at high frequency ($\sim 10^{-4}$ Hz), d is the thickness of perovskite film, A is active area. Furthermore, the charge mobility (μ) is extracted by the Mott-Gurney Law:

$$\mu = \frac{8J_D d^3}{9\varepsilon\varepsilon_0 V^2} \quad (12)$$

where J_D is the dark current density and V is the applied voltage.

Supplementary Note 7. Transient photovoltage (TPV) and transient photocurrent (TPC) analysis.

The TPV and TPC are fitted with mono-exponential decay equation:[12]

$$R_t = y_0 + B_1 e^{\left(\frac{-\tau}{\tau_1}\right)} \quad (13)$$

Where B_1 is the correlation coefficient of carrier lifetime τ_1.

Supplementary Note 8: J_{SC} and V_{OC} under different light intensity.

To further investigate the charge carrier recombination mechanisms, the performance of J_{SC} and V_{OC} under different light intensities are evaluated. The slope of V_{OC} relative to light intensity is represented as the ideality factor (n_{ID}):[13]

$$slope = \frac{n_{ID} k_B T}{q} \quad (14)$$

where q, k_B, and T denote the elementary charge, Boltzmann's constant, and Kelvin temperature.[14] the value of n_{ID} closing to 1 indicates that the charge carrier recombination in the devices is suppressed.[15]

We also analyzed the slope of α, which is an index factor related to current intensity. The value of α closing to 1 indicates the absence of double molecule recombination effect in the device.[16]

3. Supplementary Figures

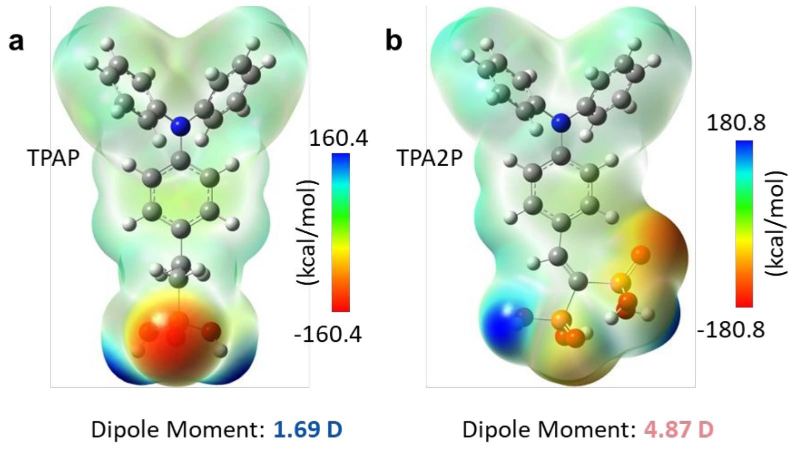

Figure S3. Electrostatic potential surfaces (EPS), and dipole moment of (a) TPAP (1.69 D) and (b) TPA2P (4.87 D) molecules.

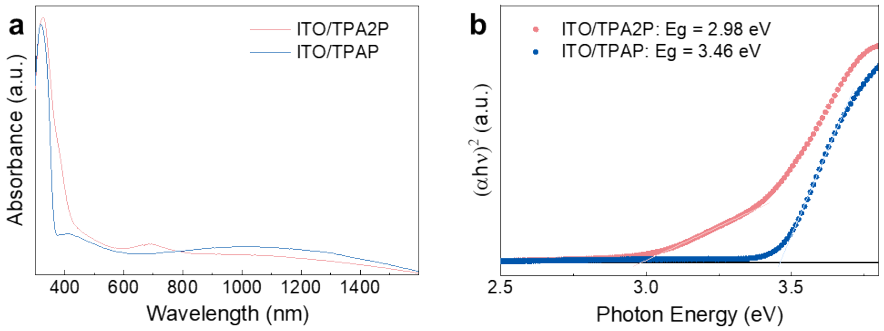

Figure S4. (a) UV-vis spectra and (b) Tauc Plot of ITO/TPAP and ITO/TPA2P films. The optical gap of ITO/TPAP and ITO/TPA2P films are 3.46 and 2.98 eV, respectively.

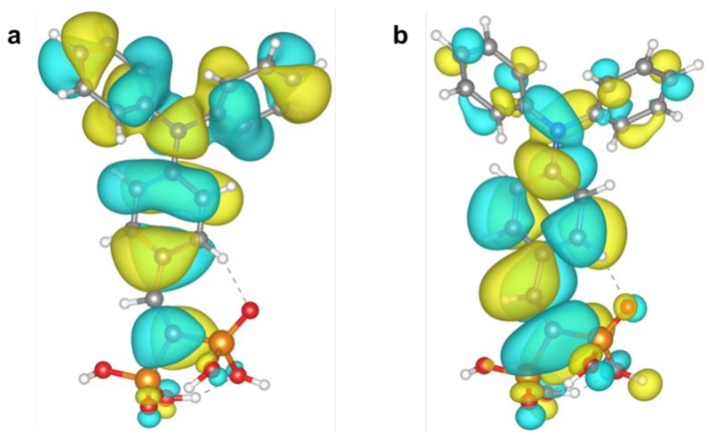

Figure S5. (a) HOMO and (b) LUMO for TPA2P molecule.

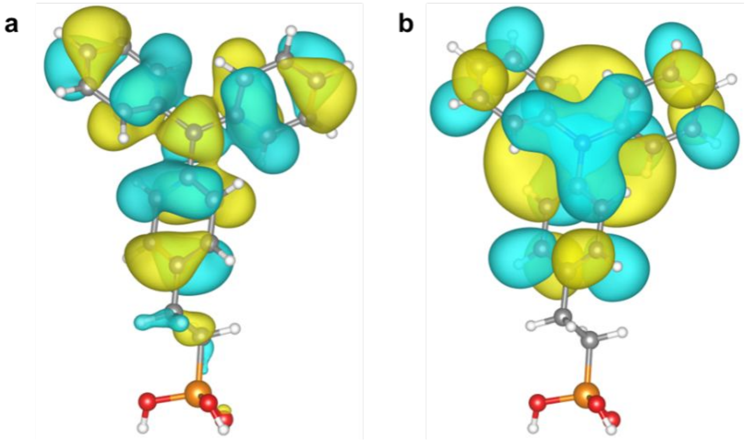

Figure S6. (a) HOMO and (b) LUMO for TPAP molecule.

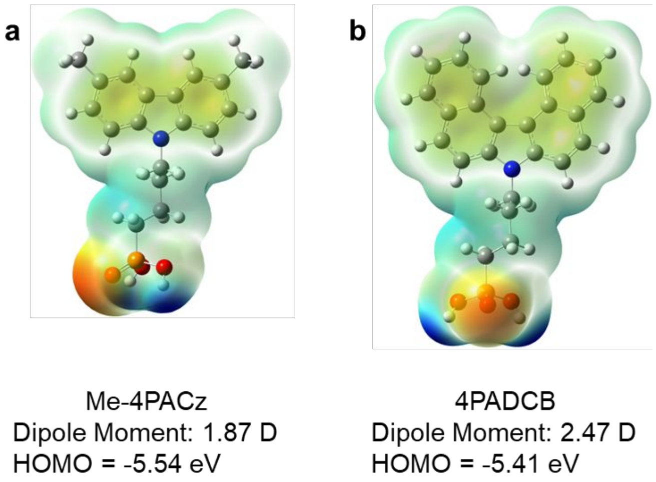

Figure S7. EPS distribution and dipole moment of (a) Me-4PACZ (1.87 D) and (b) 4PADCB (2.47 D) molecules.

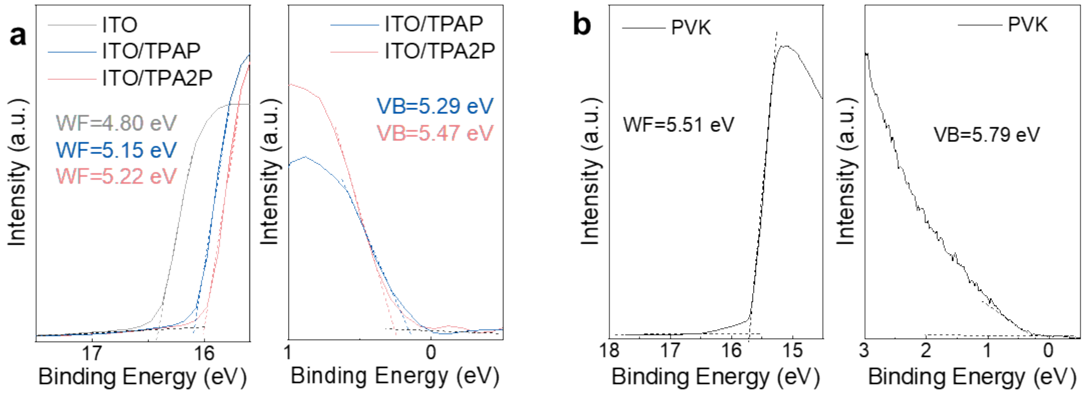

Figure S8. (a) UPS spectra of bare ITO substrate and ITO substrates covered by TPAP and TPA2P. (Left) UPS spectra around the secondary electron cutoff (WF, work function); (right) UPS spectra in the valence band (VB) region. (b) UPS spectra of buried Perovskite film. (Left) UPS spectra around the secondary electron cutoff (WF, work function); (right) UPS spectra in the valence band (VB) region.

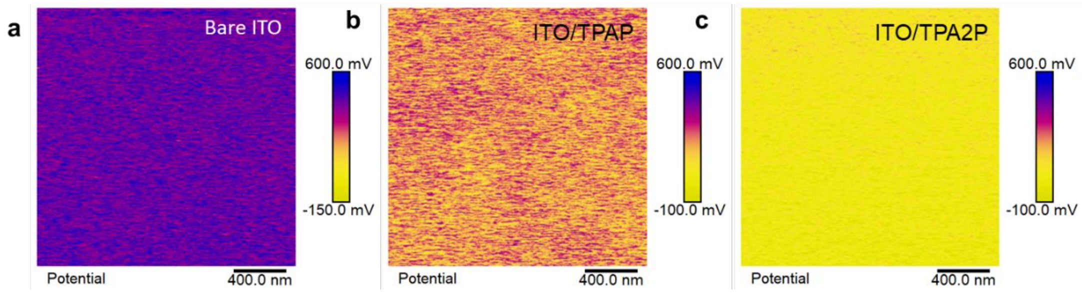

Figure S9. Surface potential images obtained by scanning Kelvin probe microscopy of (a) bare ITO, (b) TPAP and (c) TPA2P deposited on ITO surface.

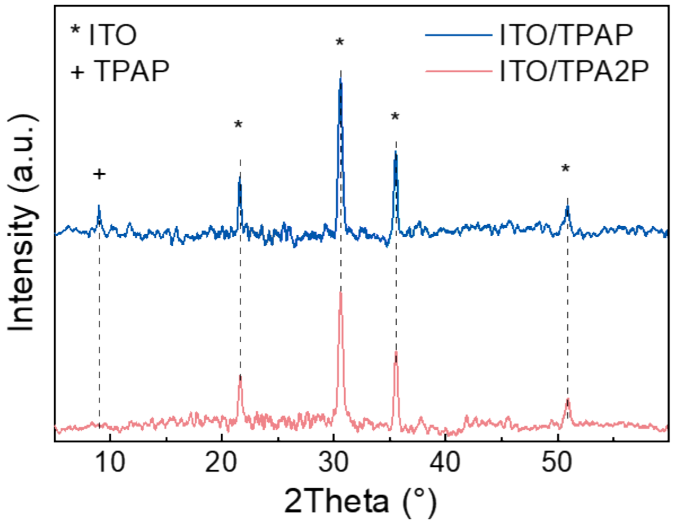

Figure S10. XRD pattern of ITO/TPAP and ITO/TPA2P films.

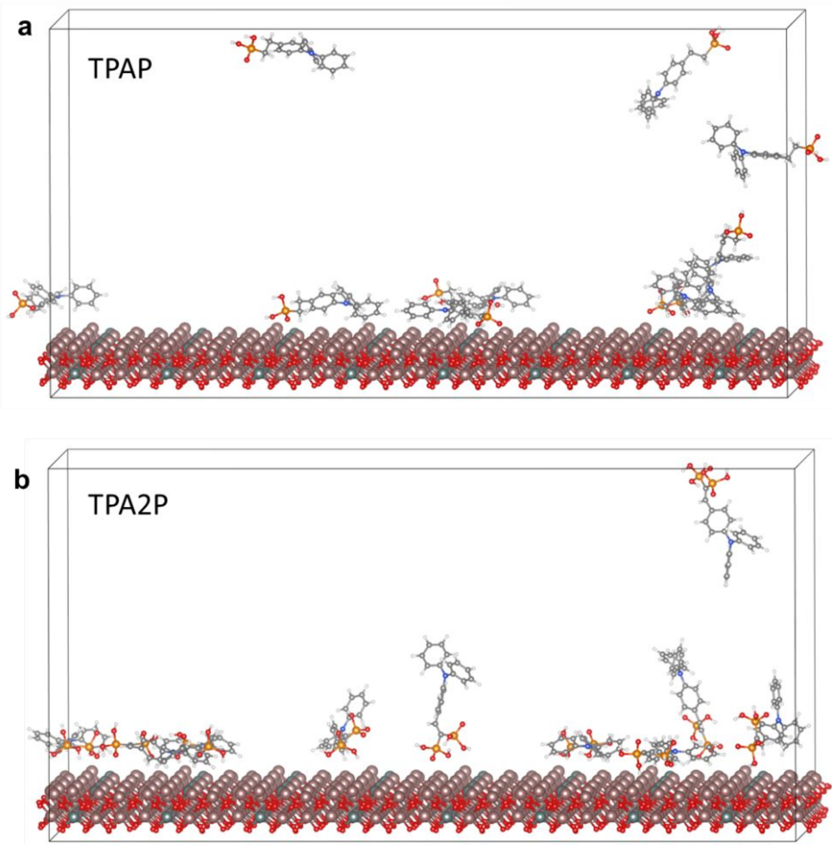

Figure S11. The adsorption distribution of 10 (a) TPAP and (b) TPA2P molecules on ITO substrate simulated by molecular dynamics method.

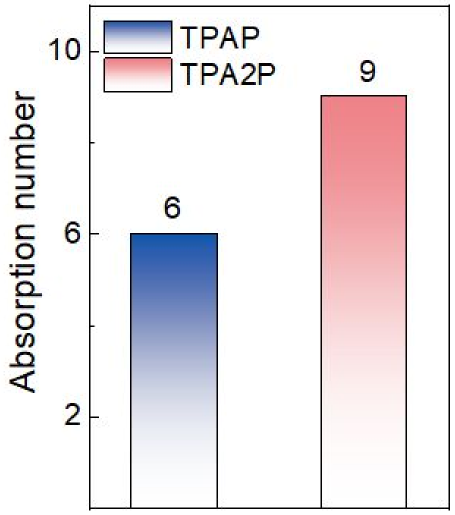

Figure S12. Adsorption number of 10 TPAP and TPA2P molecules on ITO substrate simulated by molecular dynamics method.

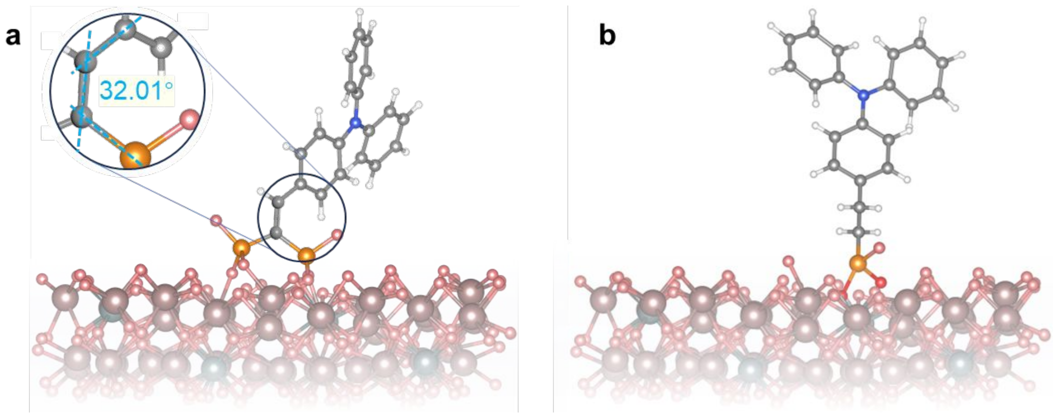

Figure S13. (a) Adsorption Configuration of the TPA2P molecules on ITO surface. The dihedral angle of the conjugated C=C double bond is 32.01°. (b) Adsorption Configuration of the TPAP molecules on ITO surface.

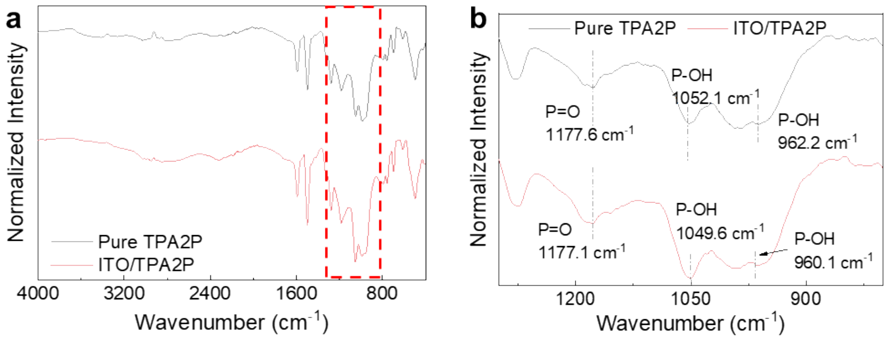

Figure S14. (a) Full FTIR spectra of pure TPA2P and ITO/TPA2P. (b) An enlarged view of the red box in Figure a, showing the interaction of a phosphate group with ITO surface.

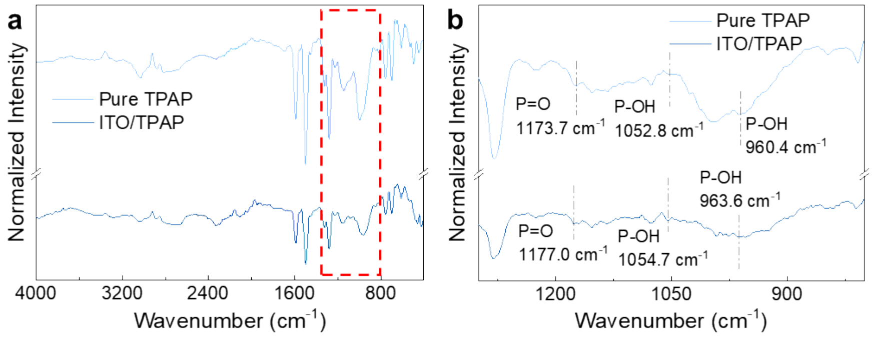

Figure S15. (a) Full FTIR spectra of pure TPAP and ITO/TPAP. (b) An enlarged view of the red box in Figure a, showing the interaction of a phosphate group with ITO surface.

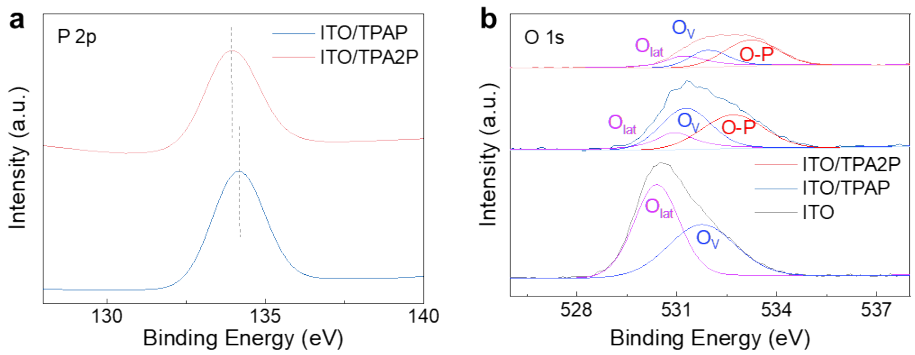

Figure S16. (a) The P 2p XPS spectra of ITO/TPAP and ITO/TPA2P. (b) The O 1s XPS spectra of bare ITO, ITO/TPAP and ITO/TPA2P.

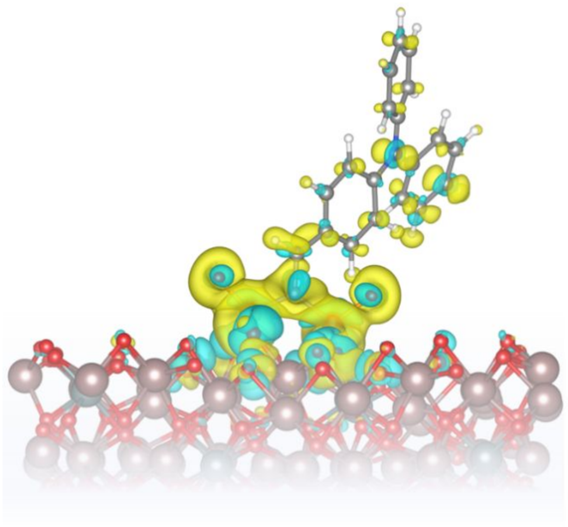

Figure S17. (a) The 3D visual charge density difference for the TPA2P molecule on ITO surface. Electron depletion/accumulation is depicted by the bule/yellow isosurfaces. at ±0.002 |e| $Å^{-3}$.

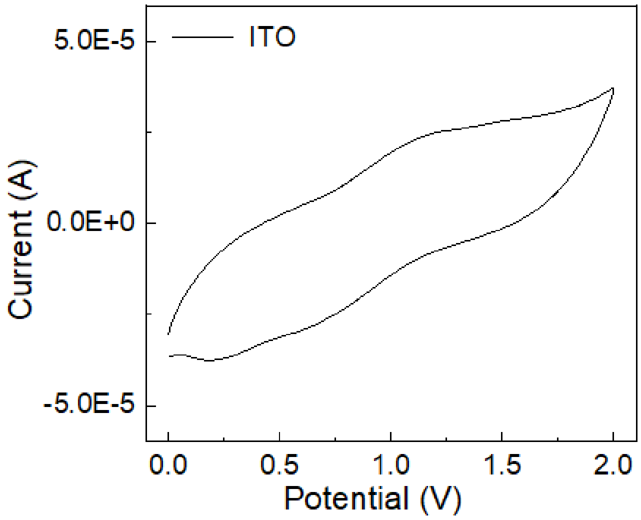

Figure S18. (a) The cyclic voltammograms (CV) curve of bare ITO.

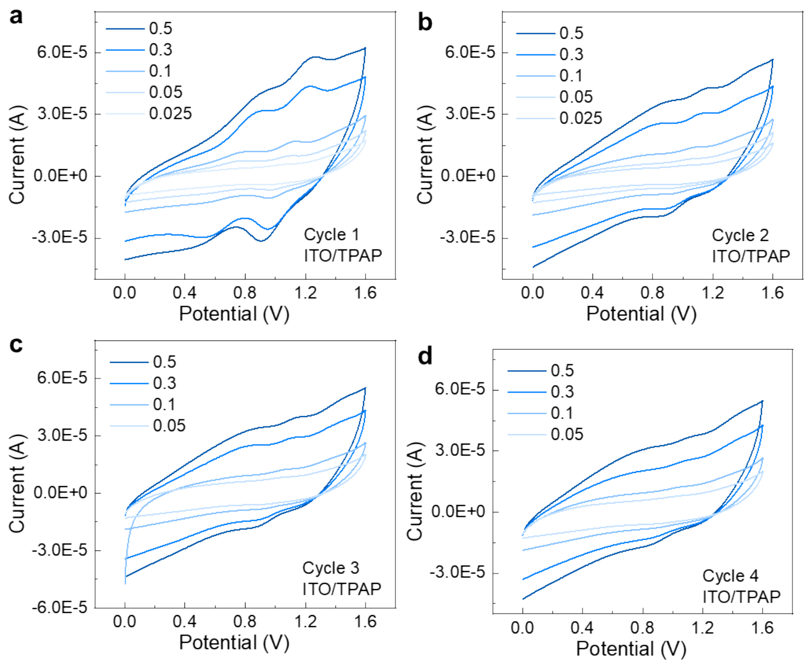

Figure S19. (a-d) The Cyclic voltammograms (CV) spectra of ITO/TPAP measured in o-DCB solution under different scan rates (V/s) and cycles. Traverse different scan rates from 0.5 V/s to 0.025 V/s is called one cycle. (a) Cycle 1; (b) Cycle 2; (c) Cycle 3; (d) Cycle 4.

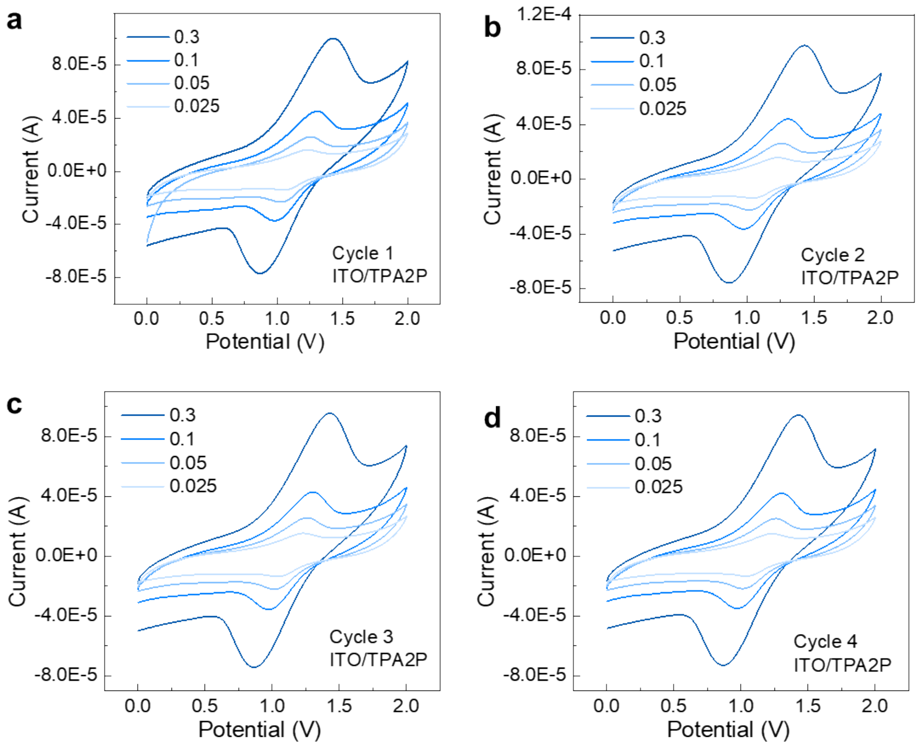

Figure S20. The CV spectra of ITO/TPA2P measured in o-DCB solution under different scan rates (V/s) and cycles. Traverse different scan rates from 0.3 V/s to 0.025 V/s is called one cycle. (a) Cycle 1; (b) Cycle 2; (c) Cycle 3; (d) Cycle 4.

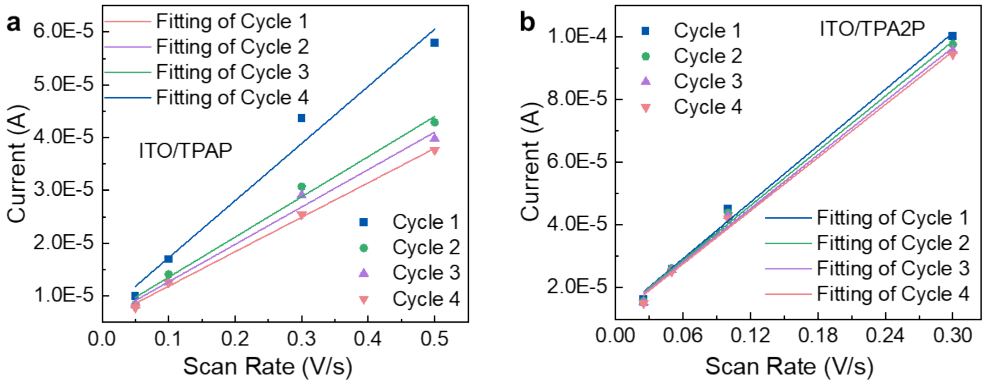

Figure S21. The corresponding peak current *vs.* scan rate chart under increased scan cycles extracted from CV curves. (a) ITO/TPAP; (b) ITO/TPA2P.

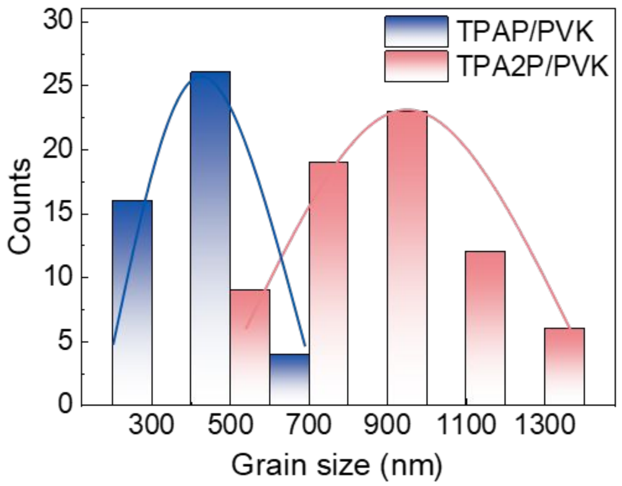

Figure S22. The SEM grain size statistical distributions of the perovskite films deposited on ITO/TPAP and ITP/TPA2P substrates.

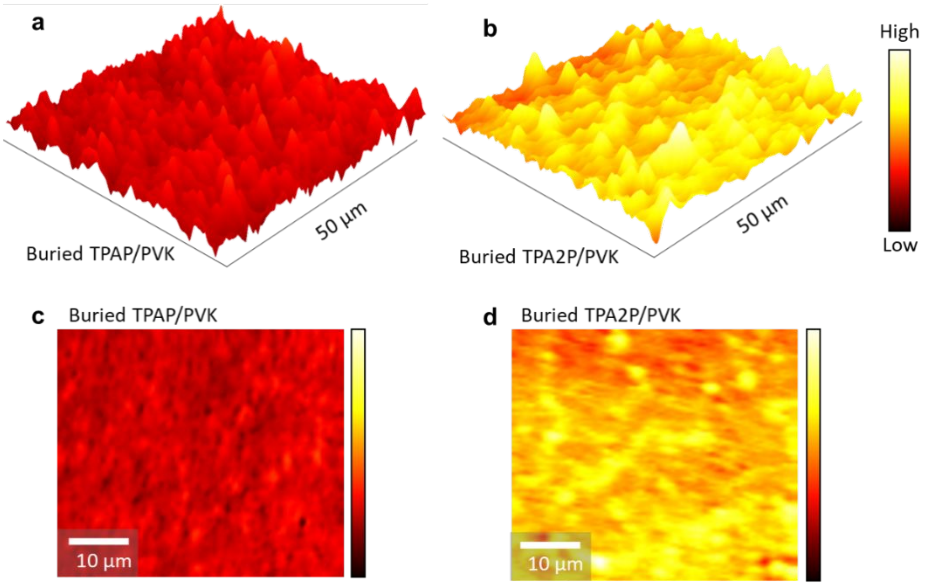

Figure S23. 3D PL mapping of buried perovskite films deposited on (a) ITO/TPAP and (b) ITP/TPA2P substrates. Top view PL mapping of buried perovskite surface deposited on (c) ITO/TPAP and (d) ITP/TPA2P substrates.

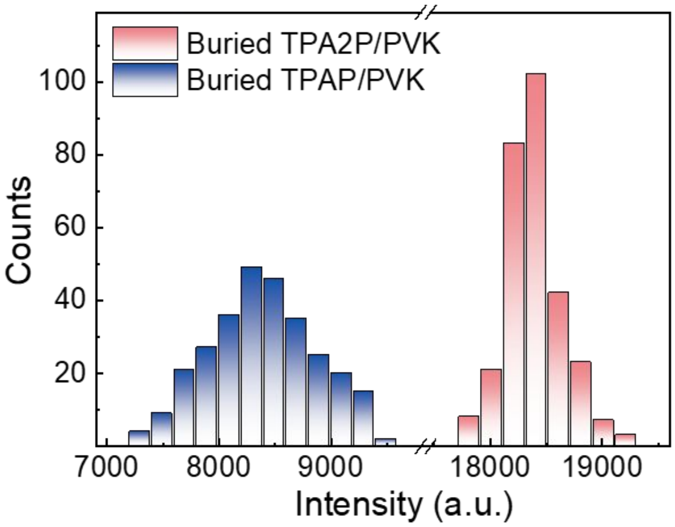

Figure S24. The PL intensity distribution of buried perovskite films deposited on ITO/TPAP and ITP/TPA2P substrates.

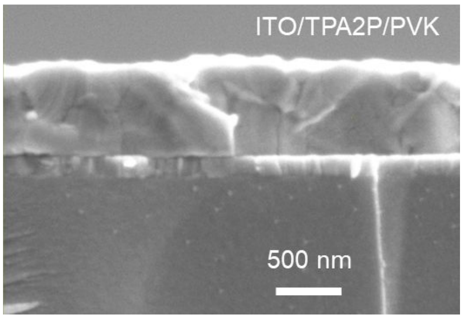

Figure S25. The cross-sectional SEM image of the ITO/TPA2P/PVK film.

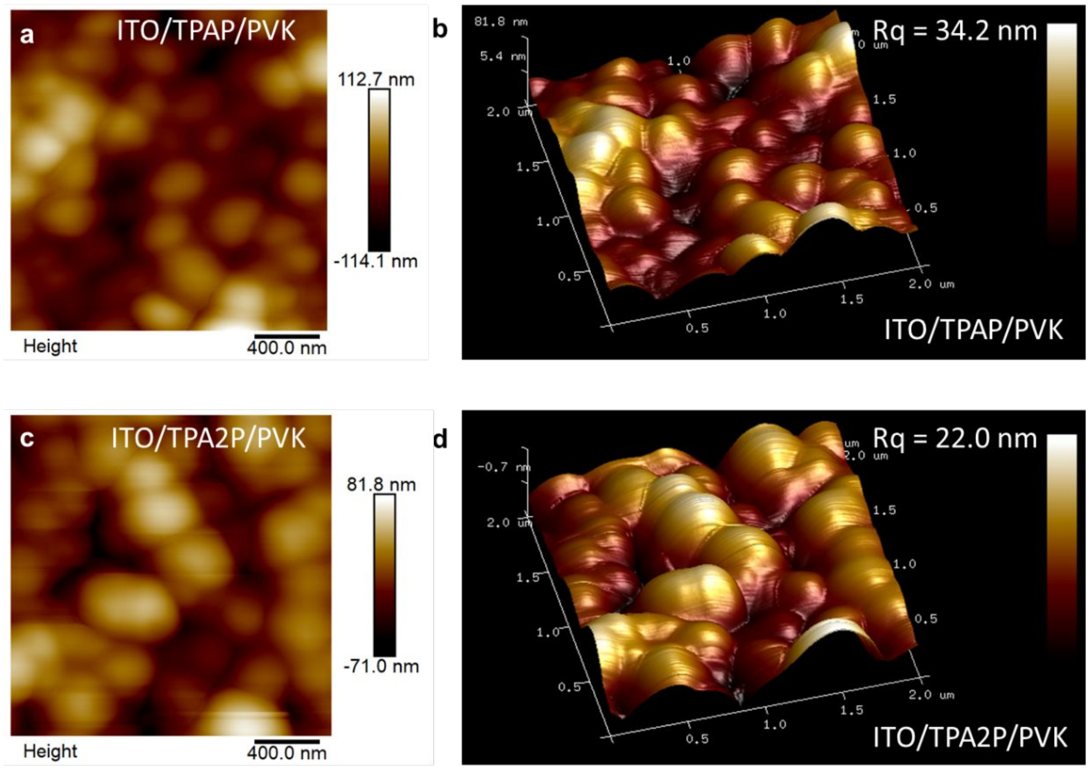

Figure S26. Atomic force microscopy (AFM) images of perovskite films deposited on (a-b) ITO/TPAP and (c-d) ITP/TPA2P substrates.

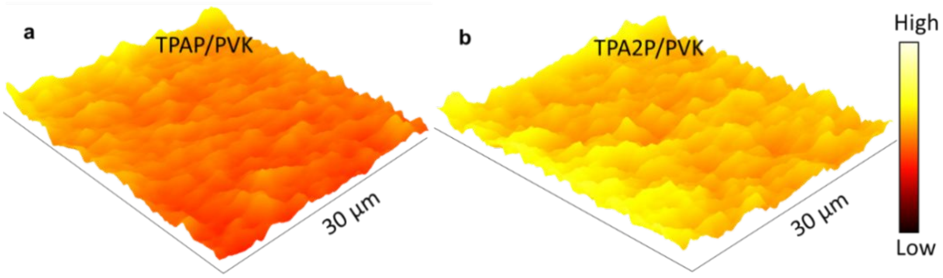

Figure S27. 3D PL mapping of top view perovskite films deposited on (a) ITO/TPAP and (b) ITP/TPA2P substrates.

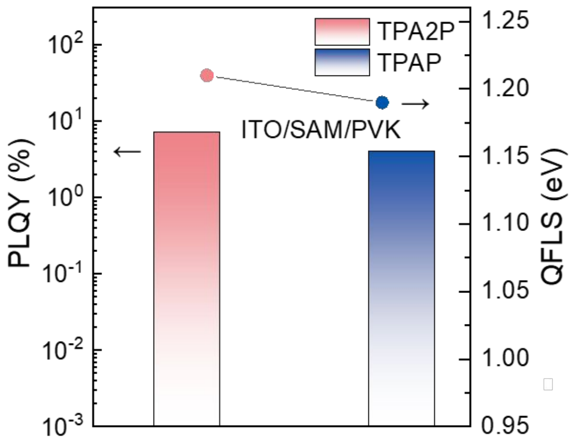

Figure S28. (a) PLQY and QFLS of perovskite films deposited on different substrates.

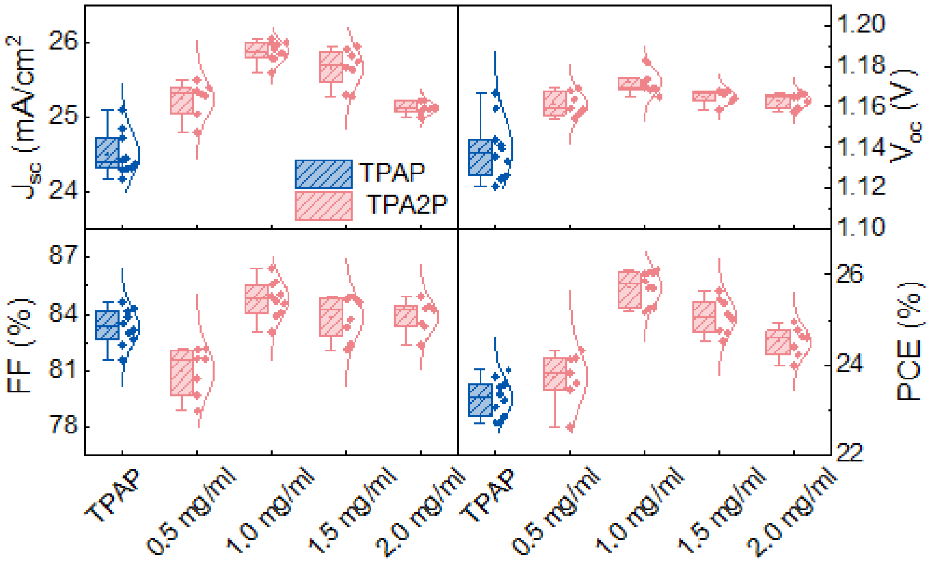

Figure S29. Statistics of device performance parameters for PSCs with optimal TPAP solution concentration and different TPA2P solution concentrations ranged from 0.5 mg/ml to 2.0 mg/ml.

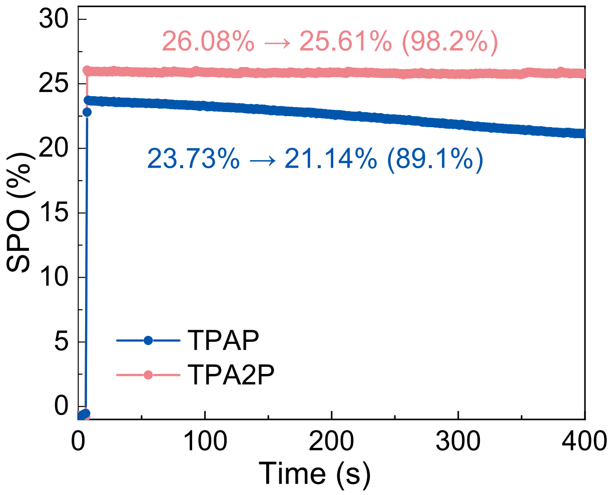

Figure S30. Stabilized power output characteristics of the TPAP and TPA2P based devices.

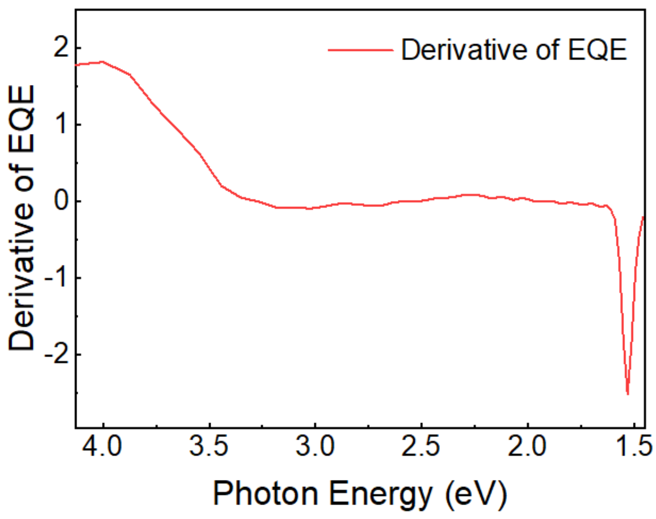

Figure S31. d(EQE)/dE versus wavelength for TPA2P based devices.

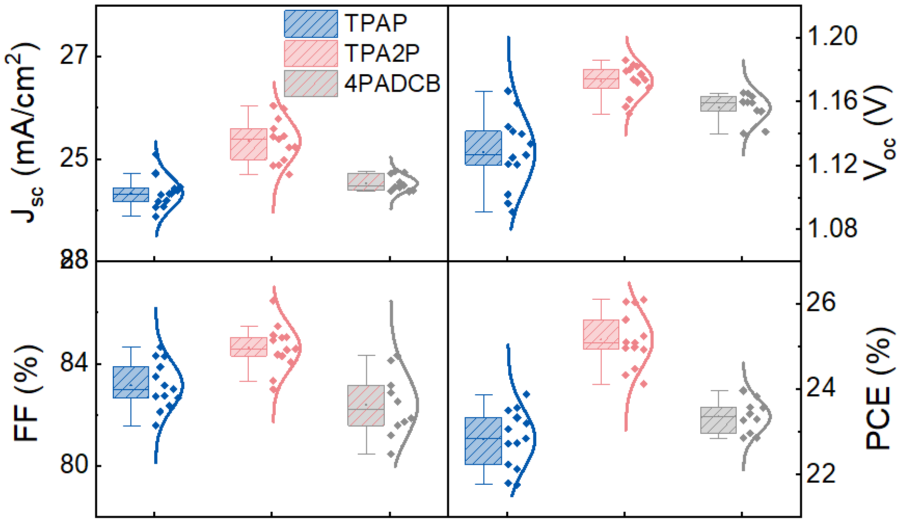

Figure S32. Statistics of device performance parameters for PSCs with different SAM layers.

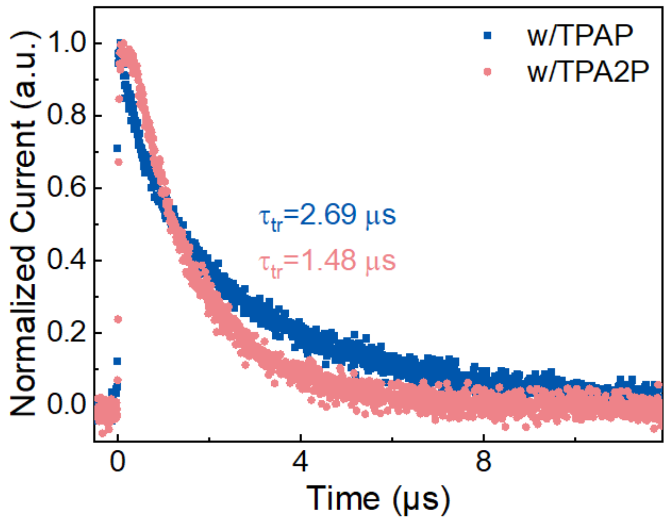

Figure S33. Transient photocurrent (TPC) carrier recombination lifetime.

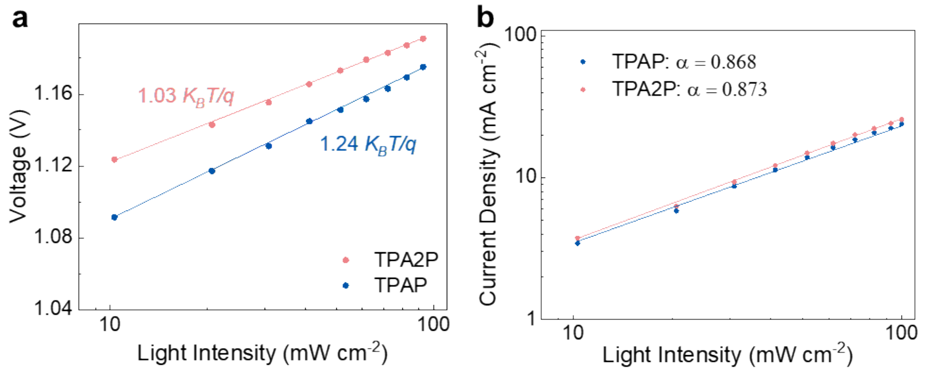

Figure S34. Light intensity-dependent measurements of (a) V_{OC} and (b) J_{SC} of the TPAP and TPA2P based devices.

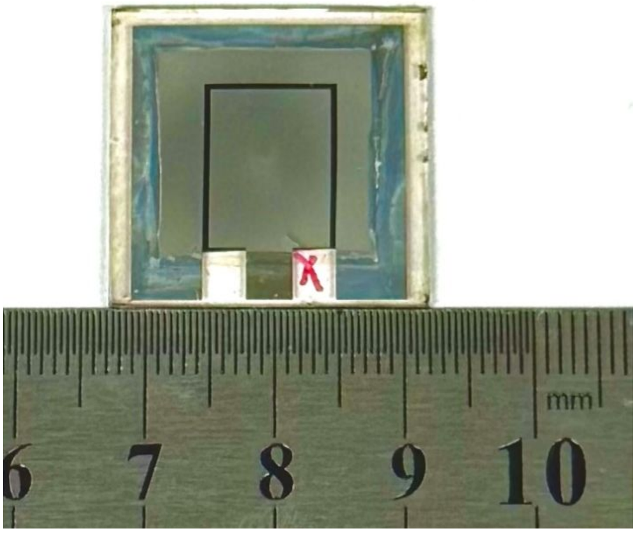

Figure S35. Photograph of large area PSCs (1.008 cm^2).

Table S1. O 1s peak areas of ITO/TPA2P and ITO/TPAP samples measured by XPS.

**Substrates**	**In-O-P/ H**	**O_V**	**O_{lat}**
ITO/TPA2P	27556.17<br>(49.9%)	14075.93<br>(25.5%)	13634.18<br>(24.5%)
ITO/TPAP	41566.52<br>(39.5%)	44743.50<br>(42.4%)	18956.82<br>(18.0%)

Table S2. The P/In atomic ratio of ITO/TPA2P and ITO/TPAP samples measured by XPS.

**Substrates**	**P 2p Atomic Concentration (%)**	**In 3d Atomic Concentration (%)**	**P/In Atomic Ratio**
ITO/TPAP	2.32	8.79	0.26
ITO/TPA2P	2.45	5.09	0.48

Table S3. Peak Areas of C 1s and In 3d for the ITO/TPAP and ITO/TPA2P films analyzed by XPS to determine their coverages. The influence of atmospheric carbon contamination has been removed.

Substrates	In 3d Area	C 1s Area	Carbon Atom Number	Coverage Factor
ITO/TPAP	730366.56	1229527.24	20	0.03
ITO/TPA2P	4074241.34	842137.23	20	0.24

Table S4. Summary of the parameters and carrier lifetimes derived from TRPL decay traces.

**Substrates**	A_1	τ_1	A_2	τ_2	τ_{avg}
TPA2P/PVK	907.01	13.98	412.37	260.78	234.75
TPAP/PVK	835.15	19.66	447.75	186.71	159.29

Table S5. Parameters summary of PLQY, QFLS and V_{OC} loss.

**Substrates**	**PLQY (%)**	**QFLS (eV)**	**V_{OC} loss (mV)**
TPA2P/PVK	7.0	1.21	59.5
TPAP/PVK	4.0	1.19	83.2

Table S6. Key Parameters summary comparing TPA2P with TPAP, 4PADCB, Me-4PACz.

Key Parameters	TPA2P	TPAP	4PADCB	Me-4PACz
Dipole Moment (D)	4.87	1.69	2.47	1.87
HOMO (eV)	-5.66	-5.38	-5.41	-5.54
Coverage Factor	0.24	0.03	/	/
Surface Density (molecules cm^{-2})	1.96×10^{14}	4.63×10^{13}	/	/
Water Contact Angle (°)	52.4	26.9	/	/
PVK Precursor Contact Angle (°)	41.0	53.5	/	/
PCE (%)	26.11	23.88	23.83	/
FF (%)	85.03	81.58	84.32	/
V_{OC} (V)	1.18	1.17	1.15	/
J_{SC} (mA/cm^2)	25.99	25.09	24.49	/

REFERENCES

(1) Kühne, T. D.; Iannuzzi, M.; Del Ben, M.; Rybkin, V. V.; Seewald, P.; Stein, F.; Laino, T.; Khaliullin, R. Z.; Schütt, O.; Schiffmann, F.; Golze, D.; Wilhelm, J.; Chulkov, S.; Bani-Hashemian, M. H.; Weber, V.; Borštnik, U.; Taillefumier, M.; Jakobovits, A. S.; Lazzaro, A.; Pabst, H.; Müller, T.; Schade, R.; Guidon, M.; Andermatt, S.; Holmberg, N.; Schenter, G. K.; Hehn, A.; Bussy, A.; Belleflamme, F.; Tabacchi, G.; Glöß, A.; Lass, M.; Bethune, I.; Mundy, C. J.; Plessl, C.; Watkins, M.; VandeVondele, J.; Krack, M.; Hutter, J., CP2K: An electronic structure and molecular dynamics software package - Quickstep: Efficient and accurate electronic structure calculations. *J. Chem. Phys.* **2020,** *152* (19), 194103.

(2) van Setten, M. J.; Giantomassi, M.; Bousquet, E.; Verstraete, M. J.; Hamann, D. R.; Gonze, X.; Rignanese, G. M., The PseudoDojo: Training and grading a 85 element optimized norm-conserving pseudopotential table. *Comput. Phys. Commun.* **2018,** *226*, 39-54.

(3) Lu, T., A comprehensive electron wavefunction analysis toolbox for chemists, Multiwfn. *J. Chem. Phys.* **2024,** *161* (8), 082503.

(4) Lu, T.; Chen, F., Multiwfn: A multifunctional wavefunction analyzer. *J. Comput. Chem.* **2012,** *33* (5), 580-592.

(5) Neese, F., Software update: The ORCA program system—Version 5.0. *WIRES COMPUT MOL SCI* **2022,** *12* (5), e1606.

(6) Hu, Q.; Rezaee, E.; Xu, W.; Ramachandran, R.; Chen, Q.; Xu, H.; EL-

Assaad, T.; McGrath, D. V.; Xu, Z.-X., Dual Defect-Passivation Using Phthalocyanine for Enhanced Efficiency and Stability of Perovskite Solar Cells. *Small* **2021,** *17* (1), 2005216.

(7) Qu, G.; Khan, D.; Yan, F.; Atsay, A.; Xiao, H.; Chen, Q.; Xu, H.; Nar, I.; Xu, Z.-X., Reformation of thiophene-functionalized phthalocyanine isomers for defect passivation to achieve stable and efficient perovskite solar cells. *J. Energy Chem.* **2022,** *67*, 263-275.

(8) Stolterfoht, M.; Caprioglio, P.; Wolff, C. M.; Márquez, J. A.; Nordmann, J.; Zhang, S.; Rothhardt, D.; Hörmann, U.; Amir, Y.; Redinger, A.; Kegelmann, L.; Zu, F.; Albrecht, S.; Koch, N.; Kirchartz, T.; Saliba, M.; Unold, T.; Neher, D., The impact of energy alignment and interfacial recombination on the internal and external open-circuit voltage of perovskite solar cells. *Energ. Environ. Sci.* **2019,** *12* (9), 2778-2788.

(9) Zhou, W.; Tai, S.; Li, Y.; Fu, H.; Zheng, Q., Achieving High-Quality Perovskite Films with Guanidine-Based Additives for Efficient and Stable Methylammonium-Free Perovskite Solar Cells. *Adv. Funct. Mater.* **2024,** *34* (46), 2407897.

(10) Zhumekenov, A. A.; Saidaminov, M. I.; Haque, M. A.; Alarousu, E.; Sarmah, S. P.; Murali, B.; Dursun, I.; Miao, X.-H.; Abdelhady, A. L.; Wu, T.; Mohammed, O. F.; Bakr, O. M., Formamidinium Lead Halide Perovskite Crystals with Unprecedented Long Carrier Dynamics and Diffusion Length. *ACS. Energy. Lett.* **2016,** *1* (1), 32-37.

(11) Brus, V. V.; Kyaw, A. K. K.; Maryanchuk, P. D.; Zhang, J., Quantifying interface states and bulk defects in high-efficiency solution-processed small-molecule solar cells by impedance and capacitance characteristics. *Prog. Photovolt: Res. Appl.* **2015,** *23* (11), 1526-1535.

(12) Zhu, P.; Wang, D.; Zhang, Y.; Liang, Z.; Li, J.; Zeng, J.; Zhang, J.; Xu, Y.; Wu, S.; Liu, Z.; Zhou, X.; Hu, B.; He, F.; Zhang, L.; Pan, X.; Wang, X.; Park, N.-G.; Xu, B., Aqueous synthesis of perovskite precursors for highly efficient perovskite solar cells. *Science* **2024,** *383* (6682), 524-531.

(13) Liu, J.; Chen, J.; Xie, L.; Yang, S.; Meng, Y.; Li, M.; Xiao, C.; Zhu, J.; Do, H.; Zhang, J.; Yang, M.; Ge, Z., Alkyl Chains Tune Molecular Orientations to Enable Dual Passivation in Inverted Perovskite Solar Cells. *Angew. Chem. Int. Ed.* **2024,** *63* (30), e202403610.

(14) Riedel, I.; Parisi, J.; Dyakonov, V.; Lutsen, L.; Vanderzande, D.; Hummelen, J. C., Effect of Temperature and Illumination on the Electrical Characteristics of Polymer–Fullerene Bulk-Heterojunction Solar Cells. *Adv. Funct. Mater.* **2004,** *14* (1), 38-44.

(15) Cao, Q.; Li, Y.; Zhang, H.; Yang, J.; Han, J.; Xu, T.; Wang, S.; Wang, Z.; Gao, B.; Zhao, J.; Li, X.; Ma, X.; Zakeeruddin, S. M.; Sha, W. E. I.; Li, X.; Grätzel, M., Efficient and stable inverted perovskite solar cells with very high fill factors via incorporation of star-shaped polymer. *Sci. Adv.* **2021,** *7* (28), eabg0633.

(16) Qu, G.; Dong, L.; Qiao, Y.; Khan, D.; Chen, Q.; Xie, P.; Yu, X.;

Liu, X.; Wang, Y.; Chen, J.; Chen, X.; Xu, Z.-X., Dopant-Free Phthalocyanine Hole Conductor with Thermal-Induced Holistic Passivation for Stable Perovskite Solar Cells with 23% Efficiency. *Adv. Funct. Mater.* **2022,** *32* (41), 2206585.
